# NS2 Protein of Hepatitis C Virus Interacts with Structural and Non-Structural Proteins towards Virus Assembly

**DOI:** 10.1371/journal.ppat.1001278

**Published:** 2011-02-10

**Authors:** Costin-Ioan Popescu, Nathalie Callens, Dave Trinel, Philippe Roingeard, Darius Moradpour, Véronique Descamps, Gilles Duverlie, François Penin, Laurent Héliot, Yves Rouillé, Jean Dubuisson

**Affiliations:** 1 Inserm U1019, CNRS UMR8204, Center for Infection & Immunity of Lille (CIIL), Institut Pasteur de Lille, Université Lille Nord de France, Lille, France; 2 Institute of Biochemistry of the Romanian Academy, Bucharest, Romania; 3 Institute of Interdisciplinary Research, University Lille 1, Villeneuve d'Ascq, France; 4 INSERM U966, Université François Rabelais and CHRU de Tours, Tours, France; 5 Division of Gastroenterology and Hepatology, Centre Hospitalier Universitaire Vaudois, Lausanne, Switzerland; 6 Laboratoire de Virologie, Centre Hospitalier Universitaire d'Amiens, Amiens, France; 7 Institut de Biologie et Chimie des Protéines, UMR-5086-CNRS, Université de Lyon, Lyon, France; Nationwide Children's Hospital, United States of America

## Abstract

Growing experimental evidence indicates that, in addition to the physical virion components, the non-structural proteins of hepatitis C virus (HCV) are intimately involved in orchestrating morphogenesis. Since it is dispensable for HCV RNA replication, the non-structural viral protein NS2 is suggested to play a central role in HCV particle assembly. However, despite genetic evidences, we have almost no understanding about NS2 protein-protein interactions and their role in the production of infectious particles. Here, we used co-immunoprecipitation and/or fluorescence resonance energy transfer with fluorescence lifetime imaging microscopy analyses to study the interactions between NS2 and the viroporin p7 and the HCV glycoprotein E2. In addition, we used alanine scanning insertion mutagenesis as well as other mutations in the context of an infectious virus to investigate the functional role of NS2 in HCV assembly. Finally, the subcellular localization of NS2 and several mutants was analyzed by confocal microscopy. Our data demonstrate molecular interactions between NS2 and p7 and E2. Furthermore, we show that, in the context of an infectious virus, NS2 accumulates over time in endoplasmic reticulum-derived dotted structures and colocalizes with both the envelope glycoproteins and components of the replication complex in close proximity to the HCV core protein and lipid droplets, a location that has been shown to be essential for virus assembly. We show that NS2 transmembrane region is crucial for both E2 interaction and subcellular localization. Moreover, specific mutations in core, envelope proteins, p7 and NS5A reported to abolish viral assembly changed the subcellular localization of NS2 protein. Together, these observations indicate that NS2 protein attracts the envelope proteins at the assembly site and it crosstalks with non-structural proteins for virus assembly.

## Author Summary

Hepatitis C virus (HCV) causes major health problems worldwide. Understanding the major steps of the life cycle of this virus is essential to developing new and more efficient antiviral molecules. Virus assembly is the least understood step of the HCV life cycle. Growing experimental evidence indicates that, in addition to the physical virion components, the HCV non-structural proteins are intimately involved in orchestrating morphogenesis. Since it is dispensable for HCV RNA replication, the non-structural viral protein NS2 is suggested to play a central role in HCV particle assembly. Molecular interactions between NS2 and other HCV proteins were demonstrated. Furthermore, NS2 was shown to accumulate over time in endoplasmic reticulum-derived structures and to colocalize with the viral envelope glycoproteins and viral components of the replication complex in close proximity to the HCV core protein and lipid droplets. Importantly, specific mutations within NS2 that affected HCV infectivity could also alter the subcellular localization of NS2 protein and its interactions, suggesting that this subcellular localization and its interactions are essential for HCV particle assembly. Altogether, these observations indicate that NS2 protein plays an important role in connecting different viral components that are essential for virus assembly.

## Introduction

The hepatitis C virus (HCV) has a high propensity to establish a persistent infection in the human liver. Approximately 170 million people suffer from chronic hepatitis C and are at risk to develop cirrhosis and hepatocellular carcinoma [1]. Current antiviral therapy is based on the use of polyethylene glycol conjugated interferon alpha in combination with ribavirin. However, this treatment is expensive, relatively toxic and effective in only approximately half of the treated patients [2]. A better understanding of the HCV life cycle is therefore essential for the development of more efficacious and better tolerated anti-HCV treatments.

HCV is an enveloped virus that belongs to the *Hepacivirus* genus in the *Flaviviridae* family [3]. HCV has a positive strand RNA genome encoding a single polyprotein that is cleaved by cellular and viral proteases into 10 different proteins: core, E1, E2, p7, NS2, NS3, NS4A, NS4B, NS5A and NS5B [3]. The non-structural proteins NS3 to NS5B are involved in the replication of the viral genome, whereas the structural proteins (core, E1 and E2) are the components of the viral particle (reviewed in [4]). The remaining proteins, p7 and NS2, are dispensable for RNA replication and there is no evidence that they are part of the viral particle [5], [6].

For reasons still unknown, HCV clinical isolates do not propagate in cell culture. However, with the development of a cell culture system that enables a relatively efficient amplification of HCV (HCVcc) [7], [8], [9], all the steps of the HCV life cycle can be investigated. Due to the accumulation of HCV core protein around lipid droplets (LDs) (reviewed in [10]), a role of these lipid bodies in HCV assembly has been suspected for a long time. Moreover, it has recently been shown that viral non-structural proteins like NS5A and NS3 and double stranded viral RNA are also present around LDs [11], [12]. The association between core and LDs seems to play a role in the recruitment of the other viral proteins and for virus production [12], [13]. Furthermore, NS5A plays a double role in both replication and assembly processes as a potential switch between these two steps [14], [15], [16], [17].

Since it is dispensable for HCV RNA replication and it does not seem to be incorporated into viral particles [6], NS2 has been suspected to be involved in the assembly process of HCV particle. Recently, experimental evidence supporting this hypothesis has been obtained [18], [19], [20]. Although the role of NS2 in the assembly process remains elusive, some data suggest that NS2 might interact with viral partners involved in virion morphogenesis. Indeed, construction of chimeric viruses between different genotypes identified the C-terminus of the first transmembrane segment of NS2 as the optimum crossover point [21]. Thus, a genetic interaction was implied between the N-terminus of NS2 and the upstream structural proteins. In the context of a chimeric virus containing genotype 1a and 2a sequences, adaptive mutations in E1, p7, NS2 and NS3 were identified, also suggesting genetic interactions between these proteins [22]. Moreover, a detailed rescue mutant analysis recently showed genetic interactions between NS2, E1E2 and NS3-4A [23]. However, despite these genetic analyses we have almost no understanding about NS2 interactions with other viral proteins and the role of these interactions in the production of infectious particles. Here, we report molecular interactions between NS2 and p7 and E2 proteins. Using a functional HCVcc virus with a reporter epitope at the N-terminus of NS2, we found that NS2 accumulates in dotted structures derived from the endoplasmic reticulum (ER) and colocalizes with E1, E2, NS3 and NS5A in close proximity to the core protein and LDs. Mutations and deletions in the p7-NS2 region affecting the subcellular localization of NS2 and its physical protein-protein interactions abolished viral assembly. Moreover, mutations in other viral proteins reported to inhibit the assembly process induced consistent changes in NS2 subcellular localization. Together, these data suggest that p7, NS2 and E2 form a functional unit which drives the proteins in the proximity of the LDs where NS2 crosstalks with other viral proteins during the virion assembly process.

## Results

### Mutation rationale in NS2

**Figure 1:**
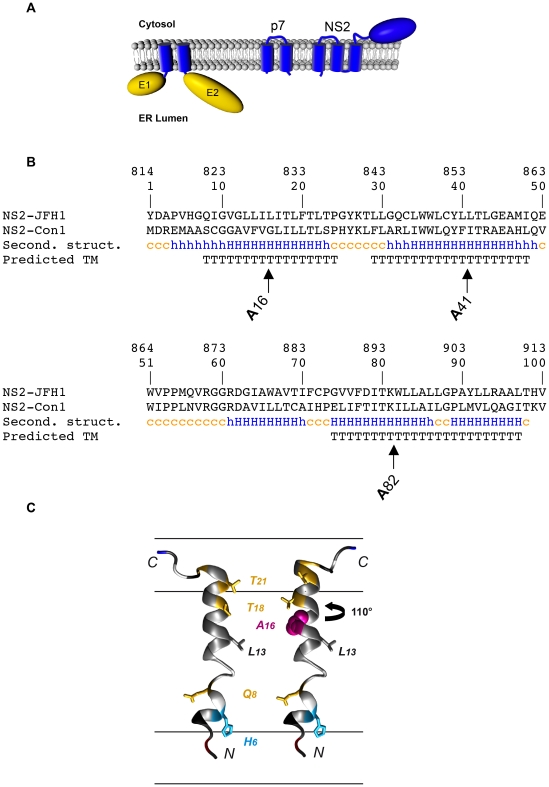
Rationale for mutagenesis in NS2 transmembrane region. (**A**) Schematic representation of the topology of E2, p7 and NS2 proteins. (**B**) Position of the inserted alanine residues in the putative N-terminal membrane domain of NS2. Alignments of NS2 membrane domain sequences from HCV strains JFH1 (genotype 2a, accession number AB047639) and Con1 (genotype 1b, AJ238799). Amino acids are numbered with respect to NS2 and the HCV JFH1 polyprotein (*top row*). *Second. struct.*, secondary structure deduced from the NMR analyses of NS2 synthetic peptides from Con1 strain [19] (Jirasko et.al. the 16^th^ international Conference on HCV and Related Viruses, Nice, October 3–7, 2009); c = coil, h = helix; capital letters indicate canonical helix structure. *Predicted TM*, consensus transmembrane (TM) segment predictions were deduced from a set of 6 available web-based algorithms prediction methods (DAS, TOPPRED2, TMHMM, SOSUI, TMPRED, PHD-TM) and represented by stretches of “T”. Arrows indicate the positions of the various alanine insertions. (**C**) Ribbon representations of the molecular homology model of NS2[1–27] of JFH1 (*left*) and the theoretical model for alanine insertion mutant A16 (*right*). An Ala insertion (shown in *magenta*) twists the helix by 110°. The N-terminal part of the model is shown in the same orientation as in the left model to highlight the distortion of residue positions on the C-terminal part of the helix. The side chains of indicated residues are shown to highlight this distortion. These models were constructed by using the NMR structure of Con1 NS2[1–27] ([19]; PDB entry, 2JY0) as template and the Swiss-PdbViewer program (http://www.expasy.ch/swissmod/). Residues are colored based on the chemical properties of their side chains: hydrophobic (*gray*) and polar (*yellow*). Acidic (Asp) and basic (Arg, Lys) residues are *red* and *blue*, respectively. His is *cyan*, and Gly is *light gray*. The membrane interfaces and hydrophobic core are schematically represented.

NS2 is a polytopic transmembrane protein containing 3 putative transmembrane segments [19](Figure 1). The p7 polypeptide and E1E2 heterodimer, which are putative partners of NS2, are also membrane proteins that contain transmembrane segments [24], [25]. Due to their respective topologies (Figure 1A), it is expected that interactions between these three proteins would involve helix-helix contacts in their transmembrane segments. Furthermore, helix-helix interactions between transmembrane segments of NS2 are also likely to take place.

To analyze the role of the transmembrane domain of NS2 in potential protein-protein interactions, we used a previously reported deletion mutant of NS2 (ΔTM12) which lacks the first two transmembrane segments [19]. For a more refined analysis, we also used alanine scanning insertion mutagenesis, a technique which has been shown to disrupt helix-helix interactions in a membrane environment [26], [27], [28], [29]. As illustrated in Figure 1C for the first transmembrane segment of NS2, this approach is based on the fact that insertion of a single amino acid into a transmembrane helix displaces the residues on the N-terminal side of the insertion by 110° relative to those on the C-terminal side of the insertion. The subsequent perturbation of the residue side-chain distribution could disrupt a potential helix-helix packing interface involving residues on both sides of the insertion. Such mutations are therefore expected to disrupt helix-helix interactions between the transmembrane segments of NS2 and/or between NS2 and other putative partners like p7 or E1E2.

Based on the NMR structure of the transmembrane segments of NS2 [19](Jirasko et al., 16^th^ International Symposium on HCV and Related Viruses, Nice, October 3–7, 2009), we designed insertion mutations in the transmembrane domain of NS2. Three different mutants were designed by inserting alanine residues close to the middle of the transmembrane segments of NS2 (Figure 1, B–C). The positions for alanine insertions were carefully chosen within the putative helical segments to preserve the overall fold of the corresponding helices. According to previous reports [26], [27], [28], [29], alanine insertions near the center of putative transmembrane helices were expected to be the most efficient to disrupt inter-helices potential interactions.

### NS2 transmembrane domain mediates E2-NS2 interaction

**Figure 2:**
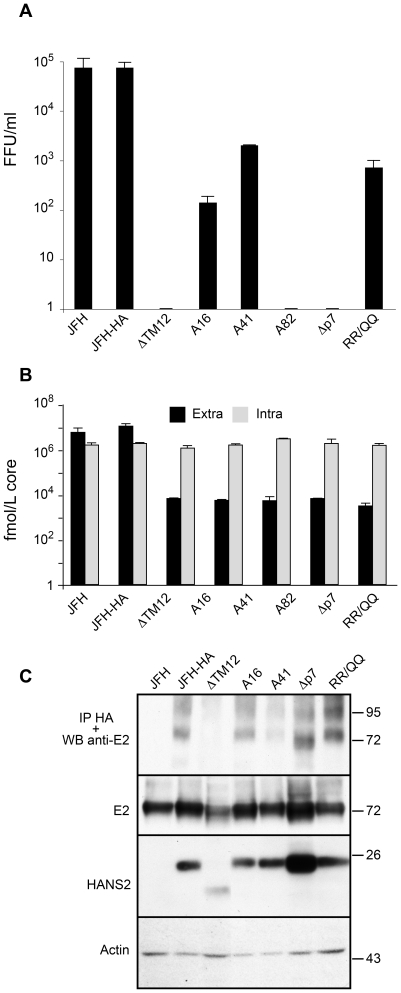
NS2 transmembrane region enables the NS2-E2 interaction. (**A**) Mutations in NS2 transmembrane region drastically decrease the production of infectious virions. Huh-7 cells were electroporated with viral RNA transcribed from different JFH-1 derived mutants. At 72h post-electroporation, the virus infectivity present in the supernatants was determined by titration of foci forming units (FFUs). Error bars indicate SD from at least two independent experiments performed in duplicate. A16, A41 and A82 correspond to JFH-HA virus with an alanine residue inserted at position 16, 41 and 82 of NS2, respectively. The following viruses were also analyzed in parallel: JFH-ΔTM12-HA (ΔTM12), JFH-Δp7-HA (Δp7) and JFH-RR/QQ-HA (RR/QQ). (**B**) The NS2 mutations affect the viral secretion, but not the replication capacity. Huh-7 cells were electroporated with viral RNA transcribed from different JFH-1 derived mutants. At 72h post-electroporation, the amount of extracellular and intracellular core antigen was determined in supernatants and cell lysates, respectively. Error bars indicate SD from at least two independent experiments performed in duplicate. (**C**) HA-NS2 co-immunoprecipitates with E2. Huh-7 cells were electroporated with viral RNA transcribed from different JFH-1 derived mutants. At 72h post-electroporation, cells were lysed and immunoprecipitation was performed with an anti-HA antibody. The immunoprecipitates were separated by SDS-PAGE and analyzed by Western blotting with an anti-E2 antibody. The presence of E2, and HA-NS2 in electroporated cells was confirmed by Western blotting and the actin content was also analyzed to verify that equal amounts of cell lysates have been loaded. It has to be noted that the E2 band corresponds to both E2 and E2p7 proteins. However, in the absence of p7, the E2-p7 form is absent.

Firstly, we wanted to evaluate the impact of our mutations on the viral life cycle. As negative controls for virus assembly, we used assembly-deficient viruses JFH-ΔE1E2-HA and JFH-Δp7-HA, containing a deletion in the regions encoding HCV envelope glycoproteins and the p7 polypeptide, respectively (Figure S1). As template for all our constructs, we used a full-length JFH1 plasmid containing adaptive mutations [30] in which the N-terminal sequence of E1 has been modified to reconstruct the A4 monoclonal antibody (Mab) epitope of the H77 isolate in order to facilitate the immunodetection of this envelope protein [31]. Moreover, we introduced an HA epitope in the N-terminus of NS2 to be used for protein detection (JFH-HA). Huh-7 cells were electroporated with different mutated viral genomes and the production of infectious virus was assessed at 72h by supernatant titration (Figure 2A). The insertion of the HA epitope did not affect the virus production as compared to the wild-type virus (JFH). As previously reported, deletion of the first two transmembrane segments prevented the production of infectious particles similar to the negative controls [19] (Figure 2A and S1). Furthermore, alanine insertions also drastically affected the viral production. While mutants A16 and A41 presented residual infectivity, A82 was not able to produce infectious particles (Figure 2A).

To identify the stage where the virus production was affected, we started with the evaluation of the replication capacity of our viruses. To this aim, we used *Renilla* luciferase reporter viruses (Figure S1A). As a negative control for replication, we used the replication-deficient construct JFH_GND_-Luc which contains a mutation in NS5B that prevents viral genome replication [7]. As the RNA input after electroporation is potentially variable, we evaluated the capacity of our viruses to replicate by determining the ratio between the luciferase activity at 72h and 4h post-electroporation when only the luciferase activity of the input RNA is present as previously shown [32]. As shown in Figure S1B, all the mutant viruses presented a similar replication capacity, which was comparable to the control viruses, whereas replication was abolished in the JFH_GND_-Luc mutant (Figure S1B-upper panel).

Since the replication was not affected, the effect of alanine insertion on NS2 protein stability was further investigated. As shown in Figure S1C, A82 insertion was detrimental for the protein integrity. For A16 mutant, the level of expression of NS2 was slightly lower as compared to the JFH-HA control. However, the level of expression of HCV proteins was higher for the JFH-HA control in this particular experiment. We therefore measured the NS2/E2 ratios which were similar for A16, A41 and JFH-HA, indicating that A16 and A41 insertions did not affect NS2 stability. Therefore, we focused our analysis on A16 and A41 mutants.

To test whether the lack of infectivity could be due to a defect in virus secretion or the release of non-infectious particles, we determined the level of core protein in the supernatants. The quantity of core protein in supernatants decreased drastically as compared to wild-type, paralleling the decrease in infectivity (Figure 2B). It has to be noted that for the mutants showing a residual infectivity (A16, A41 and RR/QQ), the release of core was at the same level as the dead mutants (ΔTM12, A82 and Δp7). This likely reflects a difference of sensitivity between the two assays. Then, we measured the viral RNA in the supernatants by qRT-PCR as previously described [17]. For all the alanine insertions, the release of viral RNA was close to the background level observed for the assembly-deficient control viruses (Figure S1B – lower panel), indicating a defect in particle secretion. These data suggest that either the process of assembly is affected at an early stage or the secretion of assembled infectious particles is impaired. To answer this question, we compared the intra and extracellular level of the core protein (Figure 2B). The ratio between intra and extracellular core was similar to JFH-Δp7-HA which was shown to be defective in early assembly steps. We also measured the intracellular infectivity of the alanine mutants in the Rluc reporter viruses context as previously reported [20]. As shown in Figure S1B (middle panel), there was no accumulation of intracellular infectivity for any of the mutants. These results suggest that mutations in the transmembrane domain of NS2 prevent the assembly process rather than the secretion of particles.

Considering the rationale of our mutagenesis, we investigated protein-protein interactions in E1E2-p7-NS2 region. Indeed, due to their position within the HCV polyprotein, it is reasonable to think that these proteins might potentially interact. Genetic and co-immunoprecipitation data suggest that NS2 interacts with E2 [23], [32], [33], [34]. Interaction assays between NS2 and E2 were performed in the context of the JFH-HA virus, which allows to immunoprecipitate NS2 with the HA tag (Figure 2C, JFH-HA). As shown in Figure 2C (JFH-HA), E2 and NS2 proteins migrated at the expected molecular mass, indicating that the polyprotein was correctly processed in these viruses. It has to be noted that in the absence of p7, E2 migrated slightly faster. This is likely due to the absence of E2-p7, which has a slightly slower migration profile in the band corresponding to E2.

The E2-NS2 interaction was then tested by co-immunoprecipitation with an anti-HA antibody followed by the detection of E2 by Western blotting. As shown in Figure 2C (JFH-HA), E2 co-precipitated with NS2, confirming that these proteins interact together in the context of the virus. In contrast, deletion of the first two transmembrane segments prevented the interaction between NS2 and E2 (Figure 2C, ΔTM12), whereas alanine insertions in the transmembrane region had different effects. While A16 (within TM1) did not affect the E2-NS2 interaction, A41 (within TM2) induced a consistent decrease in the amount of E2 co-precipitated by NS2 (Figure 2C, A16 and A41).

We further investigated the effect of p7 on the E2-NS2 interaction. To this aim, we used two mutants of p7, a deletion mutant (JFH-Δp7-HA) and a mutant having the two arginine residues in the cytosolic loop of p7 replaced by glutamine residues (JFH-RR/QQ-HA) (Figure 2C). This RR/QQ mutation is believed to abolish the ion channel activity of p7, and it induces a drastic decrease in virus production as previously reported [32], [35] and confirmed by us as shown in Figure 2A. As shown in Figure 2C (JFH-Δp7-HA and JFH-RR/QQ-HA), E2 and NS2 proteins migrated at the expected molecular mass, indicating that the polyprotein was correctly processed in these viruses. However, the two mutations had no significant effect on E2-NS2 interaction (Figure 2C JFH-Δp7-HA and JFH-RR/QQ-HA), suggesting that p7 does not modulate E2-NS2 interaction.

### NS2 accumulates in dotted structures

**Figure 3:**
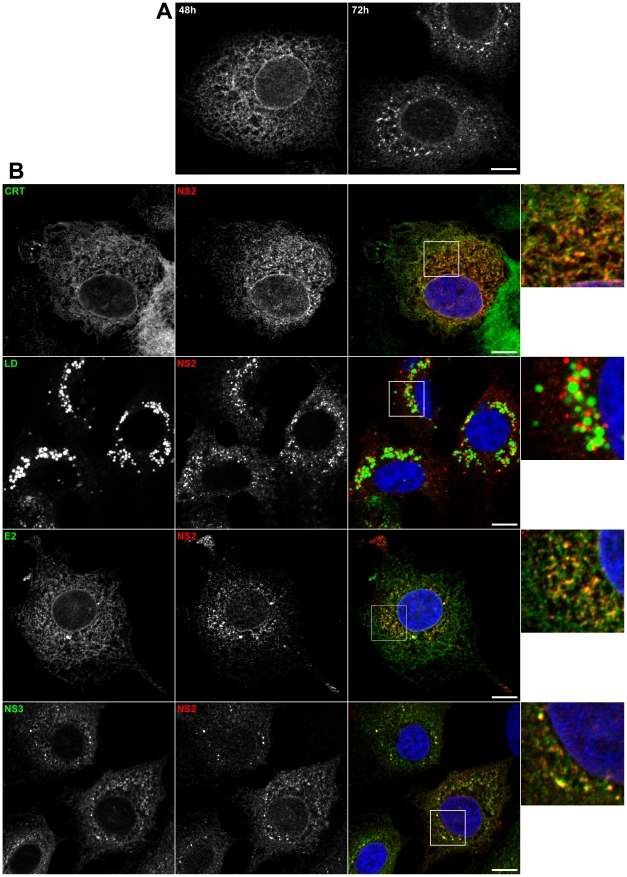
Subcellular localization of NS2. (**A**) NS2 accumulates in dotted structures. Huh-7 cells electroporated with JFH-HA RNA were grown on coverslips and fixed at 48h and 72h post-electroporation. The subcellular localization of HA-NS2 was analyzed by immunofluorescence using an anti-HA antibody. (**B**) Colocalization of NS2 dotted structures with cellular and viral markers. JFH-HA electroporated cells grown on coverslips were fixed at 72h post-electroporation and processed for double-label immunofluorescence for HA-NS2 (red) and the ER marker calreticulin (CRT in green) or the LDs stained with BODIPY 493/503 (green). JFH-HA electroporated cells were further stained for HA-NS2 (red) and HCV proteins NS3 or E2 (green). The nuclei were stained with DAPI. Representative confocal images of individual cells are shown in grey and the merge images in color. Zoomed views of the indicated areas are shown in the right column. Bar, 10 µm.

To obtain more insight into the assembly defects of our viral mutants, we decided to determine their potential effect on the subcellular localization of NS2. To this aim, we first characterized the subcellular localization of the wild-type NS2 in the context of an infectious virus, which has never been reported before. NS2 presented an ER-like reticulated pattern, and in some cells, accumulated in dotted structures (Figure 3A and data not shown). The number of these NS2-positive dot-like structures increased over time, suggesting a transition from an initial reticulate pattern to these dotted structures. At 72 hours post-electroporation, 34±15% of infected cells from 8 different electroporations displayed NS2 dots. The size of these structures was 0.84±0.38 µm (mean±SD, n = 402).

To further characterize these NS2 structures, we performed co-localization analyses with different cellular markers. As expected, the reticulate and perinuclear pattern of NS2 overlapped with calreticulin staining as well as other ER markers like calnexin and PDI (data not shown). Interestingly, NS2 dots also colocalized with ER markers (Figure 3B), but they did not colocalize with other organelle markers of the secretory pathway (data not shown). Importantly, NS2 dots were also found in close proximity of LDs (Figure 3B), suggesting that they might play a role in HCV assembly.

**Figure 4:**
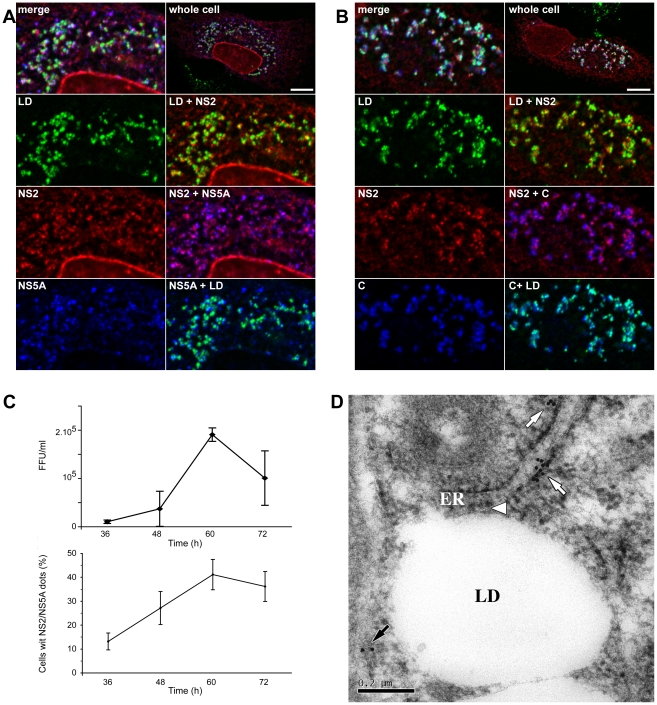
NS2 colocalization with viral proteins in association with LDs. JFH-HA electroporated cells grown on coverslips were fixed at 72h post-electroporation and processed for triple-label immunofluorescence for HA-NS2 (red), the LDs (green), and (**A**) NS5A or (**B**) core protein (C) (blue). Individual confocal images of each labeling are shown in left panels, with a merged image of the three channels in the top left panel. Two by two overlays are shown in right panels. A view of the entire cell is shown in the top right panel. Bar, 10 µm. (**C**) The kinetics of virus production for JFH-HA. Huh-7 cells were electroporated with viral RNA transcribed from JFH-HA construct. At different time points post-electroporation, the virus infectivity present in the supernatants was determined by titration of foci forming units (FFUs)(upper panel). Error bars indicate SE from at least two independent experiments performed in duplicate. The kinetics of NS2/NS5A positive dots parallels virus production (lower panel). Huh-7 cells electroporated with JFH-HA RNA were grown on coverslips and fixed at different time points post-electroporation and labeled with anti-HA and anti-NS5A antibodies. The cells which presented at least 3 dots NS2/NS5A positive were considered positive for NS2 dotted structures. The results were expressed as percentage of total counted infected cells. At least 300 infected cells were counted for each time point. Error bars indicate SE from at least two independent experiments. (**D**) NS2 detection by immuno-EM microscopy. JFH-HA electroporated cells grown in 75 cm^2^ flasks were fixed at 72h post-electroporation and processed for immuno-electron microscopy labeling with anti-HA antibody. A representative image is shown where three HA-NS2 clusters (arrows) could be observed in the proximity of a LD. Two of these clusters of gold particles (white arrows) are lying on well-preserved ER bilayers. For one of these clusters of gold particles, a connection between the ER bilayer and the LD could be observed (white arrowhead). A third cluster of gold particles (black arrow) is observed on a less preserved ER bilayer.

To better understand the potential role of NS2 dots in virus assembly, we analyzed the subcellular localization of NS2 in relationship with the other viral proteins. NS2 dots overlapped with HCV envelope glycoproteins E2 (Figure 3B) and E1 (Figure S2). NS5A and NS3 were also shown to colocalize with NS2 in its dotted pattern (Figures 3B and S2). Importantly, the structures containing both NS2 and NS5A were observed in close proximity to LDs (Figure 4A). This is illustrated by the presence of magenta dots (red NS2 and blue NS5A) in the proximity of LD (green). Finally, as observed with the LDs, NS2 dots were also found in close proximity to core protein (Figure S2), and as expected this association was observed in close proximity to LDs (Figure 4B). As shown in Figure 4B, core (blue) is tightly associated to LD (green) as the LD becomes cyan due to colocalization, NS2 (red) localizes in regions juxtaposed to the cyan LD (Figure 4B). Relying on the spatial proximity of NS2 dots, core, E1E2, NS3, NS5A proteins and LDs, we speculated that NS2 present in these structures is involved in the assembly process. Thus, we established some criteria of functionality for NS2 positive structures. They have to localize in the proximity of LDs and core protein and more importantly to colocalize with NS5A protein, which we used as a criteria for quantification purposes.

It has to be pointed out that a low number of cells contained NS2-positive structures with a different pattern of subcellular localization (Figure S3). This different pattern was indeed observed in approximately 1 to 3% of the cells at 72h post-infection or post-electroporation. These structures colocalized less with ER markers and they overlapped with ERGIC-53, a marker of the ER-to-Golgi intermediate compartment [36] (Figure S3). Furthermore, these NS2 dots were detected in close proximity to the ER exit sites, which were identified by markers of the COP II coatomer, Sec31 and p125 [37], [38] (Figure S3 and data not shown). However, cells containing these NS2 positive structures showed dramatic alterations of the secretory compartments as observed by immunofluorescence analysis of ER-to-Golgi intermediate compartment and Golgi morphology using ERGIC-53 and GM130 as markers (Figure S3). It is worth noting that NS2 did not colocalize with NS3 or NS5A and it was not found in the proximity of core and LDs in these cells (Figure S3). Due to these alterations, it is unlikely that these cells are involved in the production of infectious virus.

We asked further the relevance of NS2 dots for the production of infectious particles. After electroporation, we determined the titer of virus production at different time points (Figure 4C, upper panel). In parallel, we counted the number of cells which presented NS2/NS5A positive dots for reasons detailed above (Figure 4C, lower panel). The kinetics of virion production and the percentage of cells presenting NS2/NS5A positive dots showed a high correlation with a correlation coefficient of 0.9. To exclude the possibility that the NS2 phenotype depends on the cell culture adaptive mutations, we performed a similar experiment with a virus that does not contain the mutations. As for the adaptive mutant, virus production in the absence of mutation paralleled the NS2/NS5A dots formation with a significant correlation coefficient (data not shown). These data reinforce the idea that NS2/NS5A positive dots are involved in the virus production process.

To further investigate the NS2 localization, we performed immuno-electron microscopy with an anti-HA antibody on Huh7 cells which were electroporated with JFH-HA RNA and prepared by cryosubstitution. As shown in Figure 4D, we could identify clusters of gold particles in the proximity of LDs. Moreover, two of the clusters are lying on preserved ER bilayers. For one of the clusters of gold particles, a connection between the ER bilayer and the LDs could be observed (Figure 4D). Thus, the immuno-electron microscopy confirms the juxtaposition between NS2 dots and LDs in a 0.2µm range, which is consistent with the observations in confocal microscopy. It has to be noted that gold particles were detected on both sides of the membrane even if the HA epitope is supposed to be located in the ER lumen. This is compatible with the length of two antibodies (primary+secondary) since the gold particles were never further away than 30 nm from the membrane. However, we cannot exclude a double topology for NS2 as recently suggested [39].

### NS2 transmembrane region is crucial for its subcellular localization

**Figure 5:**
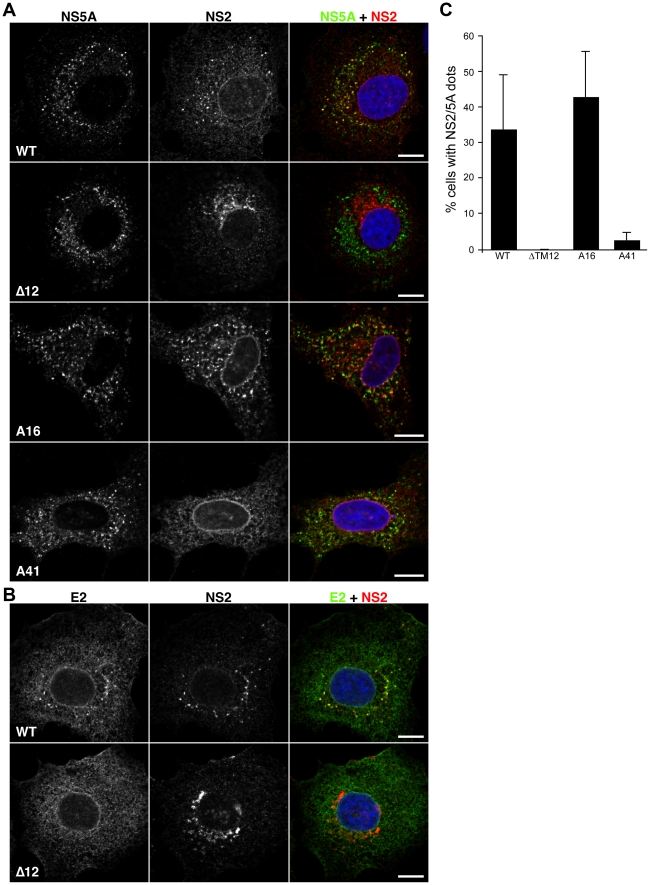
Subcellular localization of NS2 mutants in TM region. (**A**) and (B) Effect of mutations on the accumulation of NS2 in dotted structures. Huh-7 cells electroporated with JFH-HA RNA (WT) or mutant genomes were grown on coverslips and fixed at 72h post-electroporation. The following viruses were analyzed: JFH-HA (WT), JFH-?TM12-HA (?TM12), JFH-A16-HA (A16) and JFH-A41-HA (A41). The subcellular localization of HA-NS2 was analyzed by immunofluorescence using anti-HA (red), anti-NS5A (green) (Panel A) and anti-E2 (green) (Panel B) antibodies. The nuclei were stained with DAPI. Representative confocal images of NS2 and NS5A labelings are shown in grey, and the merge images in color. Bar, 10 micro µm. (C) Mutations in NS2 transmembrane region drastically decrease the number of cells presenting NS2 dotted structures. Huh-7 cells electroporated with JFH-HA RNA were grown on coverslips and fixed at different time points post-electroporation and labeled with anti-HA and anti-NS5A antibodies. Cells showing at least 3 NS2/NS5A dots were considered positive for NS2 dotted structures. The results were expressed as percentage of total counted cells. At least 250 infected cells were counted. Error bars indicate SD from at least two independent experiments.

The next obvious step was to determine the subcellular localization of NS2 for the different mutants defective in assembly. In the case of ΔTM12, NS2 localized in confined structures which did not colocalize with NS5A and they were not associated with the core protein or LD (Figure 5, panels A and C and data not shown). Moreover, there was no colocalization between the truncated NS2 and the E2 glycoprotein (Figure 5B), which correlates with the lack of interaction between the two proteins (Figure 2C). Then, we analyzed the subcellular localization of NS2 for the alanine insertion mutants. While A16 mutant presented NS2 dotted structures as wild-type, A41 mutation induced a drastic decrease in the percentage of cells with NS2 dots (Figure 5, panels A and C). These data suggest that NS2 transmembrane region is an important localization determinant.

### Core, envelope and p7 proteins influence NS2 subcellular localization

A peculiarity of the *Flaviviridae* family is the involvement of both structural and non-structural proteins in the assembly process (reviewed in [40]). Thus, we wanted to investigate the NS2 subcellular localization in the context of assembly deficient viruses having mutations in different viral proteins.

**Figure 6:**
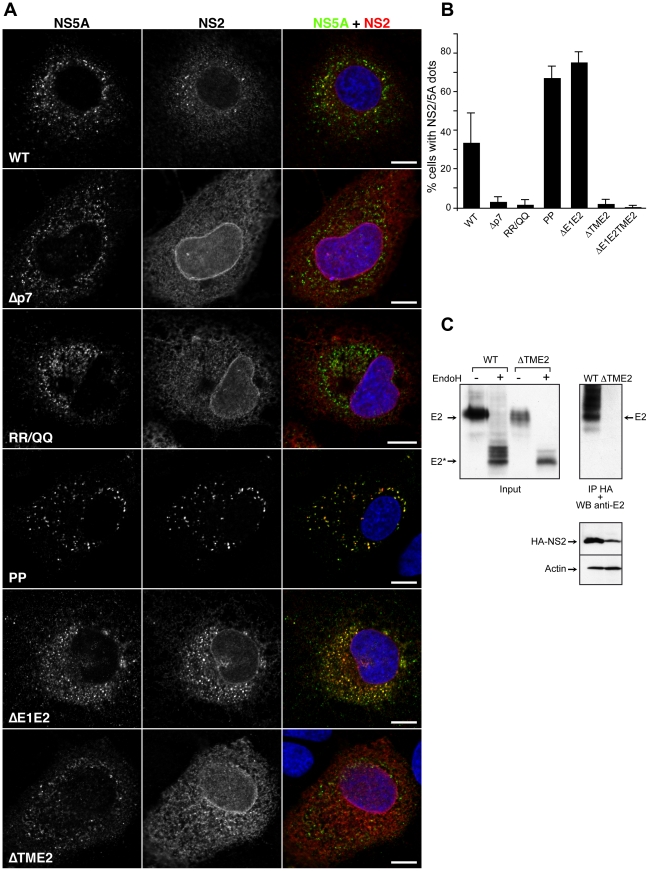
Subcellular localization of NS2 in assembly deficient mutants in the structural region. (**A**) Effect of mutations on the accumulation of NS2 in dotted structures. Huh-7 cells electroporated with wild-type or mutant genomes were grown on coverslips and fixed at 72h post-electroporation. The subcellular localization of HA-NS2 was analyzed by immunofluorescence using anti-HA (red) and anti-NS5A (green) antibodies. The nuclei were stained with DAPI. The following viruses were analyzed: JFH-HA (WT), JFH-Δp7-HA (Δp7), JFH-RR/QQ-HA (RR/QQ), JFH-HA-PP (PP), JFH-ΔE1E2-HA (ΔE1E2) and JFH-ΔTME2-HA (ΔTME2). Representative confocal images of NS2 and NS5A labelings are shown in grey, and the merge images in color. Bar, 10 µm. (**B**) Mutations in the structural region of HCV have different effects upon the number of cells presenting NS2 dotted structures. Huh-7 cells electroporated with JFH-HA RNA were grown on coverslips and fixed at different time points post-electroporation and labeled with anti-HA and anti-NS5A antibodies. Cells showing at least 3 NS2/NS5A dots were considered positive for NS2 dotted structures. The results were expressed as percentage of total counted cells. At least 220 infected cells were counted. Error bars indicate SD from at least two independent experiments. In this experiment, JFH-ΔE1E2TME2-HA (ΔE1E2TME2) construct was used in addition to the above mentioned viruses. (**C**) The transmembrane domain of E2 mediates the NS2-E2 interaction. Huh-7 cells were electroporated with viral RNA transcribed from JFH-HA or JFH-ΔTME2-HA (ΔTME2) mutant. At 72h post-electroporation, cells were lysed and immunoprecipitation was performed with an anti-HA antibody. The immunoprecipitates were separated by SDS-PAGE and analyzed by Western blotting with an anti-E2 antibody. Glycosylated or deglycosylated lysates were blotted against E2 and HA-NS2. The actin content was also analyzed to verify that equal amounts of cell lysates have been loaded.

Recruitment of core protein to LDs was reported to be essential for a productive assembly process [12], [13]. The proline residues 138 and 143 in domain D2 of the core protein are crucial for virus production and core recruitment to LDs [13], [41]. Furthermore, the mutation of these proline residues has been previously shown to prevent the core induced recruitment of NS5A to the LDs [12]. Therefore, we introduced these mutations in the context of JFH-HA virus (Figure 6, JFH-HA-PP). As previously reported [13], the mutation prevented the production of infectious virions (Figure 9A), and the core protein was not redistributed to LDs, which in turn remained spread in the cytoplasm rather than the perinuclear localization induced by a functional core protein (Figure S4). As shown in Figure 6, in the context of this mutation, NS2 protein maintained the capacity to accumulate in dotted structures that colocalized with NS5A. In contrast to the wild-type, NS2 dotted structures were not found in the vicinity of LDs in the context of the PP mutation, suggesting that NS2 does not have the signals to localize by itself around the LDs (Figure S4). Importantly, in this context, the number of cells presenting NS2 dotted structures increased tremendously in comparison to the wild-type (Figure 6B). These observations suggest that the PP mutation induces a block in the assembly process, which favors the accumulation of NS2 protein in the dotted structures.

As structural proteins, the envelope glycoproteins are involved in the assembly process [42]. The envelope proteins play a crucial role in the assembly of enveloped viruses, which can be due for some viruses to the capacity of the envelope proteins to establish lateral interactions and to generate a pushing force necessary for the budding process [43]. A deletion in the envelope region would therefore block the assembly process as shown by an in-frame deletion of 351 amino acids in the envelope proteins region [7]. In the context of our JFH-HA virus, this deletion mutant is also fully replicative, and it does not produce infectious particles (data not shown). To further understand the interplay between NS2 and HCV envelope glycoproteins, we analyzed the subcellular localization of NS2 protein in the context of the E1E2 deletion. In this context, NS2 localized in NS5A positive structures juxtaposed to the LDs and core protein (Figure 6 and S4). Interestingly, as for the JFH-HA-PP virus, the number of cells presenting NS2 dotted structures also increased in the case of JFH-ΔE1E2-HA, correlating also with a block in the assembly process favoring an accumulation of NS2 dotted structures (Figure 6B).

The deletion introduced in the envelope region is predicted to generate a chimeric protein comprising the N-terminus of E1 and the C-terminus of E2 protein. Since we introduced the A4 epitope in the N-terminus of E1, we were able to detect the subcellular localization of this small chimeric protein. It is worth mentioning that we also detected this truncated chimeric protein in NS2/NS5A dotted structures, suggesting that the E2 transmembrane domain is sufficient for the recruitment of HCV envelope proteins to NS2 dotted structures (Figure S4).

The p7 protein has been shown to be crucial for the assembly process [32], [35]. Moreover, the ion channel activity of p7 correlates with the virus assembly process since mutations predicted to abolish the ion channel activity have a strong inhibitory effect on the virus production [32], [35]. We therefore also analyzed the subcellular localization of NS2 in the context of p7 mutants corresponding to a complete deletion (JFH-Δp7-HA) or an amino acid substitution (JFH-RR/QQ-HA) previously reported to affect the assembly and release of the virus [32], [35]. The two constructs behaved as expected. While JFH-Δp7-HA produced no infectious particles, JFH-RR/QQ-HA presented a 2 log_10_ decrease in virus titers at 72h post-electroporation (Figure 2A). Importantly, the two mutants induced a drastic decrease in the number of cells presenting NS2/NS5A dotted structures (Figure 6B). Together, these data indicate that NS2 needs a functional p7 polypeptide to colocalize with NS5A in dotted structures.

Other partners than p7 are likely necessary for NS2 accumulation in dotted structures. Indeed, as shown in Figure 5, A41 and ΔTM12 mutants, which fail to interact with E2, also present a drastically reduced number of NS2 dotted structures. This suggests that NS2-E2 interaction might be crucial for NS2 subcellular localization. Thus, one additional determinant could be represented by the transmembrane domain of E2, which is most likely the interacting region with NS2 due to topological constraints. In order to check this hypothesis, we used both JFH-HA and JFH-ΔE1E2-HA constructs in which we replaced the transmembrane region of E2 with the autoprotease 2A from foot and mouth disease virus (FMDV) (JFH-ΔTME2-HA and JFH-ΔE1E2TME2-HA)(Figure S1). We first verified that a proper processing of the polyprotein mediated by FMDV 2A protease has occurred in these constructs. To check our constructs, we analyzed the molecular mass of E2 following deglycosylation with EndoH or PNGase endoglycosidases. As shown in Figure 6C, E2 from JFH-HA and JFH-ΔTME2-HA presented a similar molecular mass after deglycosylation with EndoH, suggesting that the FMDV 2A protease is functional since FMDV 2A and the transmembrane domain of E2 have similar sizes. Indeed, if FMDV 2A protease had not been functional, we would have observed a difference of 7kD corresponding to the molecular mass of unprocessed p7 (Figure 6C and data not shown). Interestingly, in contrast to the wild-type envelope protein, the truncated E2 did not interact with NS2 (Figure 6D). Furthermore, in contrast to what was observed for JFH-HA and JFH-ΔE1E2-HA (Figure 6A), NS2 protein of the ΔTME2 mutant presented an ER like pattern and the formation of NS2 dotted structures was prevented (Figure 6A and B). Similar data were also obtained with JFH-ΔE1E2TME2-HA construct (Figure 6B). Thus, it seems that p7-NS2 and the transmembrane domain of E2 form a functional unit that targets these proteins to NS5A positive structures.

### NS2 interacts with p7 in a co-immunoprecipitation assay

**Figure 7:**
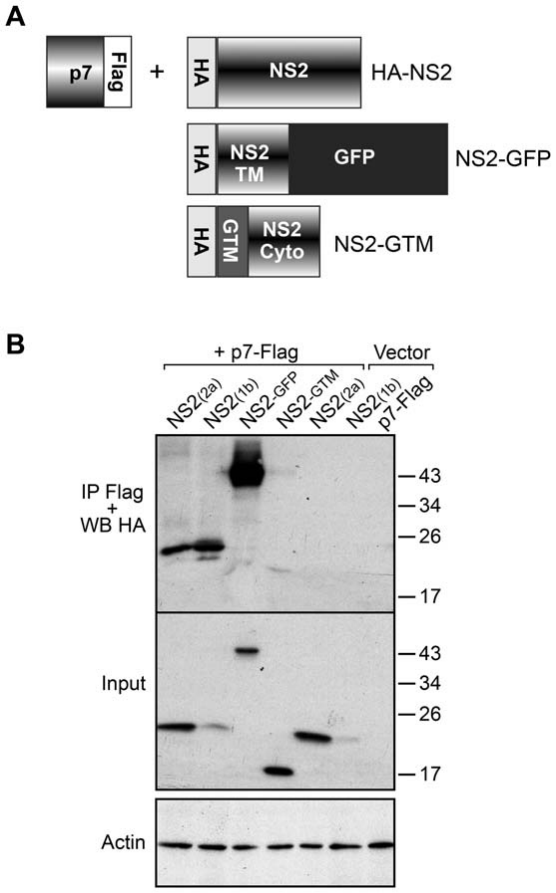
NS2 and p7 interact in a co-immunoprecipitation assay. (**A**) Schematic representation of the constructs used in this study. NS2-GFP corresponds to the transmembrane domain of NS2 in fusion with GFP, whereas NS2-GTM corresponds to the transmembrane domain of VSV-G protein in fusion with the cytosolic domain of NS2. All the proteins contain a HA tag at their N-terminus. (**B**) HA-NS2 co-immunoprecipitates with p7-Flag. 293T cells were transfected with plasmids expressing p7-Flag, HA-NS2 from different genotypes, HA-NS2 mutants or control plasmids. At 24h post-transfection cells were lysed and immunoprecipitations with an anti-Flag antibody were performed. The immunoprecipitates were separated by SDS-PAGE and analyzed by Western blotting with an anti-HA antibody to identify the presence of co-immunoprecipitated HA-NS2. The presence of HA-NS2 in transfected cells was confirmed by Western blotting. The actin content was also analyzed to verify that equal amounts of cell lysates have been used.

The above data suggest a possible interaction between p7 and NS2. We therefore explored this putative interaction in a biochemical assay, by analyzing p7-NS2 association in a co-immunoprecipitation assay. Due to the difficulties in analyzing p7-NS2 interactions in the context of an infectious virus, we analyzed these interactions by co-transfecting cells with plasmids expressing these two proteins only. In this approach, the p7 polypeptide and NS2 were tagged with a Flag or a HA epitope, respectively (Figure 7A, p7-Flag and HA-NS2).

The p7-NS2 interaction was tested after co-expression of the tagged proteins in 293T cells. Co-immunoprecipitation experiments were performed with an anti-Flag antibody linked to agarose beads. The immunoprecipitates were separated by SDS-PAGE and probed with anti-HA antibodies by Western blotting. As shown in Figure 7B, NS2 of different genotypes coprecipitated with p7. Since only p7 of genotype 1a was used in these experiments, it suggests that p7 interacts with NS2 in a genotype independent manner. However, we cannot exclude that the system is not sensitive enough to discriminate between slight changes in affinities.

Further, we constructed two chimeric proteins, NS2 tagged with a green fluorescent protein (NS2-GFP) and NS2-GTM (Figure 7A). In NS2-GFP, the cytosolic domain of NS2 was replaced by GFP protein, whereas for NS2-GTM, we replaced the transmembrane domain of NS2 by the transmembrane domain of glycoprotein G of VSV. As shown in Figure 7B, the NS2-GFP could be precipitated by p7-FLAG, while NS2-GTM could not. This clearly shows that the transmembrane region is the main determinant of p7-NS2 interaction.

### NS2 interacts with p7 in a fluorescence resonance energy transfer with fluorescence lifetime imaging microscopy (FRET-FLIM) assay

**Figure 8:**
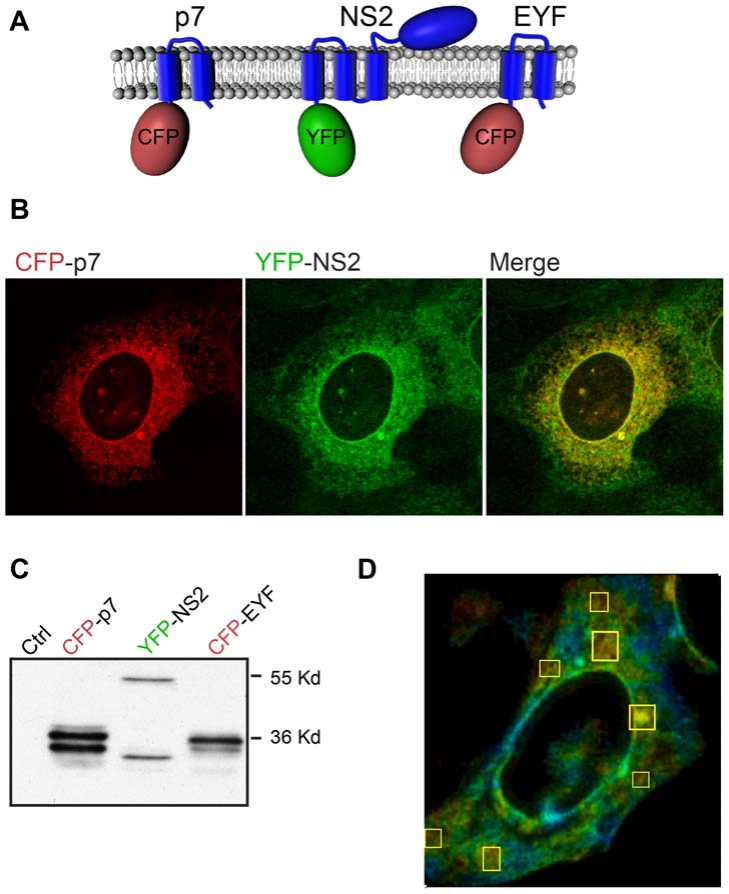
NS2 and p7 interact in FRET-FLIM analyses. (**A**) Schematic representation of the constructs used in this study. (**B**) Immunofluorescence analysis of the co-expression of CFP-p7 and YFP-NS2. U2OS cells were co-transfected with plasmids expressing CFP-p7 and YFP-NS2. At 24h post-transfection, the subcellular localization of the different proteins was assessed by confocal microscopy. (**C**) Western blot analysis of the expression of CFP-p7, YFP-NS2 and CFP-EYF. U2OS cells were transfected with plasmids expressing CFP-p7, YFP-NS2 or CFP-EYF. At 24h post-transfection, cells were lysed and protein expression was confirmed by SDS-PAGE followed by Western blotting. (**D**) FLIM analyses. Samples were subjected to FLIM and color coded maps were obtained. The regions where the FRET events are present are marked with squares. The colors represent the progression from minimum (yellow) to maximum (blue) fluorescence lifetime.

To confirm the p7-NS2 interaction with another approach, we used the FRET-FLIM technique. FRET-FLIM requires the presence of two fluorophores (a donor and an acceptor) fused in frame to the studied proteins. If the two proteins interact, an energy transfer occurs between the two fluorophores and the fluorescence life time of the donor (a parameter of the energy emitted by the donor) will decrease. To measure p7-NS2 interactions by FRET-FLIM, Cerulean fluorescent protein (CFP) and Venus yellow fluorescent protein (YFP) were fused to the N-terminus of p7 and NS2, respectively (Figure 8A).

As previously reported, CFP-p7 and YFP-NS2 showed a reticulate perinuclear distribution (Figure 8B), which is characteristic of ER proteins [34], [44]. Western blotting analyses indicated that CFP-p7 and YFP-NS2 migrate at the expected molecular mass with some degradation products of lower molecular weight (Figure 8C). The energy transfer in FRET-FLIM assay needs the integrity of the fluorophores and the correct positioning of the interacting partners. The degradation products could fall into two categories: either soluble fluorophores or membrane bound truncated chimeras. In either case the energy transfer is unlikely to occur with the cleavage products. Thus, the presence of degradation byproducts is unlikely to influence the accuracy of the FRET-FLIM acquisitions.

After biphoton laser excitation and data analysis, fluorescence life time maps were built. Interestingly, the regions showing interactions were located in distinct spots throughout the cells as illustrated in a fluorescence life time color-coded map (Figure 8D). A summary of FRET-FLIM analysis is presented in Table 1. The mean life time of fluorescence decreased from 2.69±0.12 ns (n = 10) in cells transfected with the donor only (CFP-p7) to 2.34±0.09 ns (n = 10) for double transfections (CFP-p7+YFP-NS2). The variation of the mean donor life time is characteristic for energy transfer between two fluorescent proteins in FRET-FLIM analyses as previously observed for other protein-protein interactions [45], [46]. As a positive control, we used the transmembrane domains of HCV glycoproteins E1 and E2 which are known to interact and to have the same subcellular localization as p7 and NS2 [25]. The positive control couple presented a comparable decrease in the mean lifetime of the donor to CFP-p7/YFP-NS2 couple. Indeed, the mean life time decreased from 2.56±0.03 ns (n = 14) in cells transfected with the donor only (CFP-E2) to 2.35±0.08 ns (n = 14) for double transfections (CFP-E2+YFP-E1). As a negative control, we used CFP fused to the transmembrane domain of yellow fever virus E protein (CFP-EYF), a donor protein with the same topology and localization as p7 [28], [47]. As shown in Table 1, the mean life time of the donor in monotransfection did not change in double transfections confirming the lack of interaction between CFP-EYF and YFP-NS2 (n = 11). The biphoton pictures for the positive and the negative control are shown in Figure S5. Thus, these data strongly suggest that p7 and NS2 proteins interact intracellularly.

**Table 1:** Mean values of the fluorescence lifetime.

Proteins	The mean lifetime of the donor protein (ns)
CFP-p7	2.69+/−0.12
CFP-p7+YFP-NS2	2.34+/−0.09*
CFP-E2	2.56+/−0.03
CFP-E2+YFP-E1	2.35+/−0.08*
CFP-EYF	2.59+/−0.02
CFP-EYF+YFP-NS2	2.59+/−0.06

*p<0.005. The statistical significance of the results was determined by using the Student's t-test after testing the normal distribution of the data with the Shapiro-Wilk test [68].

### NS5A stabilizes NS2 dotted structures

**Figure 9:**
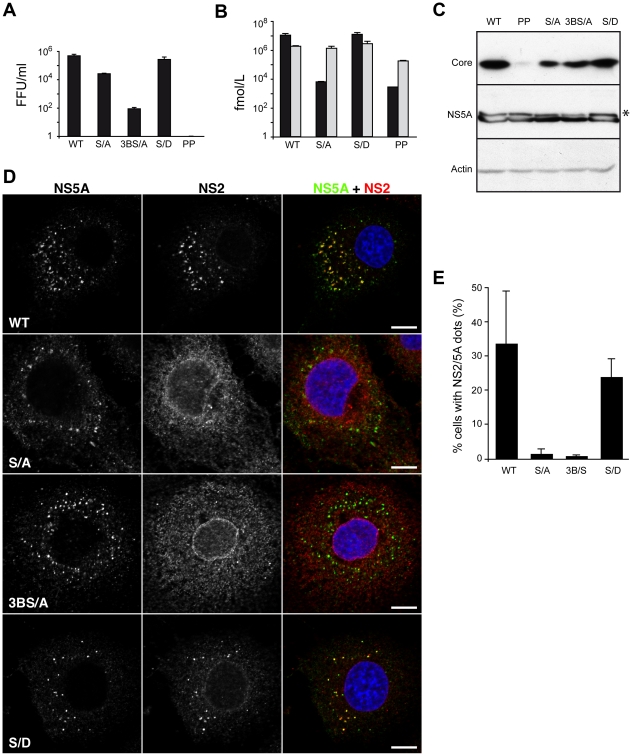
Subcellular localization of NS2 in assembly deficient mutants in NS5A protein. (**A, B**) Phenotype of the NS5A mutants. Virus infectivity (**A**), extra (black bars) and intracellular (light grey bars) core determination (**B**) were performed as described in Figure 2. The following viruses were analyzed: JFH-HA (WT), JFH-S/A-HA (S/A), JFH-3BS/A-HA (3BS/A) and JFH-S/D-HA (S/D). In addition, JFH-HA-PP (PP) was also analyzed in parallel. (**C**) Analysis of the expression and phosphorylation of the NS5A mutants. The hyperphosphorylated form of NS5A is indicated by an asterisk. The presence of core and NS5A was confirmed by Western blotting and the actin content was also analyzed to verify that equal amounts of cell lysates have been loaded. (**D**) Effect of mutations on the accumulation of NS2 in dotted structures. Huh-7 cells electroporated with JFH-HA RNA (WT) or mutant genomes were grown on coverslips and fixed at 72h post-electroporation. The subcellular localization of HA-NS2 was analyzed by immunofluorescence using anti-HA (red) and anti-NS5A (green) antibodies. The nuclei were stained with DAPI. Representative confocal images of NS2 and NS5A labelings are shown in grey, and the merge images in color. Bar, 10 µm. (**E**) Mutations in NS5A protein drastically decrease the number of cells presenting NS2 dotted structures. Huh-7 cells electroporated with JFH-HA RNA or the indicated mutants were grown on coverslips, fixed at 72 h post-electroporation and labeled with anti-HA and anti-NS5A antibodies. Cells showing at least 3 dots NS2/NS5A positive were considered positive for NS2 dotted structures. The results were expressed as percentage of total counted cells (at least 228). Error bars indicate SD from at least two independent experiments.

Among the non-structural proteins, NS5A is the most characterized in terms of its role in the assembly process. NS5A is recruited through direct interaction by the core protein around LDs where its domain III is involved in the assembly process potentially by its phosphorylation [14], [16], [17]. By deletion mutagenesis, Tellinghuisen et al. identified a cluster of serine residues at positions 452, 454 and 457, which are crucial for virus production [17]. Furthermore, by alanine scanning mutagenesis, Masaki et al. reported that the same serine cluster is involved in the direct interaction between NS5A and core protein [16]. While Tellinghuisen et al. reported that serine 457 alone is essential for virus production, Masaki et al. showed that only double mutants had a significant impact on virus production [16], [17]. The apparent contradiction might be explained by the different viruses and time points for virus production assessment. While Tellinghuisen et al. used a chimeric virus consisting of the structural proteins of J6 strain up to NS2 protein, Masaki et al. used the wild type JFH strain [16], [17]. Thus, we constructed the two mutants in the context of our JFH-HA virus – JFH-S/A-HA and JFH-3BS/A-HA, respectively. We analyzed the phenotype of the mutants as well as the polyprotein processing. The results fitted the literature with JFH-S/A-HA virus infectivity moderately reduced at 72h and JFH-3BS/A-HA profoundly impaired in infectious virus production, while the replication and protein integrity were unaltered (Figure 9A, B, C). As reported, we showed that JFH-S/A-HA and JFH-3BS/A-HA present less hyperphosphorylated NS5A (Figure 9C). Surprisingly, for both mutants, NS2 localized mainly in an ER-like pattern and the number of cells with NS2/NS5A dots decreased dramatically (Figure 9D, E). The serine 457 may be replaced by an aspartate residue, which mimics a phosphoserine [17]. We therefore introduced the same mutation (JFH-S/D-HA) and as reported the virus titers were restored at wild-type levels (Figure 9A). Interestingly, this mutant partially recovered the NS2 subcellular localization both qualitatively and quantitatively (Figure 9D, E). Together, these results suggest that NS5A phosphorylation might stabilize the NS2 dotted structures in the assembly process.

## Discussion

Our understanding of the HCV morphogenesis process is still in its infancy. Different viral components were identified as players in the morphogenesis process. As expected, the structural proteins are essential in the virus makeup [7]. The scenario gets more complicated with the involvement of non-structural proteins in the assembly process. The p7 polypeptide, NS2, NS3, NS4B, NS5A were reported to be involved in viral assembly [14], [17], [20], [22], [32], [48]. However, the mechanism of the complex interplay between the structural and non-structural proteins towards the virion production is not understood. In this paper, we provide evidence for molecular interactions between NS2, p7 and E2, respectively. Furthermore, we show that NS2 accumulates over time in ER-derived dotted structures and colocalizes with the envelope glycoproteins and components of the replication complex in close proximity to the core protein and LDs. Characterized assembly deficient mutants in both structural and non-structural proteins present qualitative and quantitative modifications in NS2 subcellular localization. Indeed, specific mutations within NS2, p7 or E2 modify the subcellular localization of NS2 and impair virus production. Mutations in core, envelope proteins or NS5A affect the NS2 subcellular localization along with the virus titers. Altogether, these observations indicate that NS2 protein crosstalks with both structural and non-structural proteins during virus assembly.

Our data demonstrate a physical interaction between NS2 and p7. This interaction correlates with the previously reported genetic interactions present in the C-NS2 region [21], [22]. The HCV p7 polypeptide is a viroporin involved in viral assembly [20], [32], [49]. Viroporins represent a class of viral proteins that are involved in the viral morphogenesis process in different and largely unknown manners. Alphavirus 6K interacts with E1 and p62 envelope glycoproteins and is involved in optimal assembly and release of the virion by an unknown mechanism [50], [51]. The E protein of coronaviruses interacts with the M protein and is crucial for the assembly of virus-like particles and virions [52], [53], [54]. To our knowledge, the HCV p7 polypeptide is the first viroporin which interacts with a non-structural protein (NS2) and this might be a peculiarity of the members of the *Flaviviridae* family where the non-structural proteins are involved in particle assembly [40].

Topologically, the transmembrane domain of NS2 (NS2TM) would be the main region available for interactions with the upstream transmembrane proteins p7 and E1E2. Based on this assumption, we wanted to characterize NS2 interactions with p7 and E2 by deletion and chimeric mutants. Both NS2-E2 interaction and NS2-p7 interaction were mapped in the transmembrane region of NS2 as expected. However, since the lack of p7 does not affect the E2-NS2 interaction, we could imagine that NS2 uses separate domains to interact with p7 and the envelope proteins. Interestingly, when we deleted the transmembrane region of E2, the NS2-E2 interaction was impaired and the NS2 subcellular localization changed. Altogether, these observations suggest that p7, the transmembrane domain of NS2 and the transmembrane domain of E2 contain signals which act synergistically to direct the NS2 protein towards the NS5A positive membranes in the LD proximity.

The drastic effects of alanine insertion mutagenesis on HCV infectivity reflect more intrinsic effects on NS2 function in virus assembly. As shown for lactose permease, single alanine residue insertions into transmembrane helices of a polytopic membrane protein can be highly disruptive to protein structure and function due to their effects on intramolecular helix-helix interactions [26]. Furthermore, within the same protein, different transmembrane helices can have differential sensitivities to single residue insertions [26]. In the case of NS2, our data indicate that an insertion in the third putative transmembrane helix strongly reduces the stability of the protein, suggesting a drastic alteration of NS2 structure by this mutation. The decrease in infectivity for the mutations in the first two transmembrane helices also indicates a drastic alteration in NS2 function that can be linked to local alteration of its structure, as suggested by the change in subcellular localization of NS2 mutant A41. Finally, the drastic decrease in infectivity of mutant A16 in spite of NS2 localization in dotted structures indicates that the subcellular localization of NS2 in these structures is not sufficient by itself for infectivity. Rather, it likely needs to play additional function(s) at the site of virus assembly and such function(s) would be disrupted by the A16 mutation. One explanation could be that a weak E2-NS2 interaction as seen for A41 is not able to direct the p7-NS2 unit to the LDs and a potential strong interaction as for A16 does not allow the p7-NS2 unit to release the envelope proteins heterodimer to the assembly site and the subsequent recycling/dissociation of the unit.

Over time, a substantial part of NS2 accumulates in dotted structures localized in the ER in close proximity to the core protein which is associated to LDs. Since the LD/ER interface is considered as the potential particle assembly site [12], NS2 localization close to the LDs is expected to correlate with its function in a late step of the viral life cycle. Interestingly, NS2 accumulation in dotted structures parallels the colocalization of NS2 with NS5A, NS3 proteins and most likely the replication complex. Moreover, the NS2 dotted structures are juxtaposed to the core protein and colocalize with the envelope proteins E1 and E2. Thus the NS2 dots contain all the assembly players and are located in the microenvironment of the LDs, the proposed virus assembly site. It is worth mentioning that the formation of NS2 dotted structures is not due to a non-specific effect of the viral genome replication since some of our mutants showing the same replication rate had very different subcellular localizations of NS2 (e.g. A16 vs. A41). It seems rather that the formation of NS2 related dots represents a transition rather than an end state in a productive assembly process. Indeed, mutations in core (JFH-HA-PP) or in the envelope proteins (JFH-ΔE1E2-HA) induced an obvious increase in the number of cells presenting NS2 dots. This could mean that the p7-NS2-E1E2 complexes pre-exist in the NS5A positive subcompartment. In addition, the core-mediated redistribution of LDs could induce the recruitment of the assembly components to LDs followed by the envelope protein incorporation into the virion during the budding process. Finally, after budding, NS2 might relocate to another subcellular compartment or be degraded. This scenario is supported by the fact that NS2 dots accumulate around the LDs and core protein when the envelope proteins are deleted, which is expected to inhibit the lateral interactions between the envelope proteins [42], preventing the budding process to occur.

For the moment, the connection between NS2 and the replication complex (RC) is just inferred from genetic data and colocalization in immunofluorescence experiments [23], [34](this report). We show here that mutations in NS5A, which are reported to affect the phosphorylation status of the protein, abolish the accumulation of NS2 in dotted structures. Moreover, we could partially restore the phenotype by an aspartate mutant, which mimics phosphoserine. Thus, NS2 colocalization with NS5A is favored by the phosphorylation state of the latter. It is possible that NS5A charged state enhances a potential NS2-RC interaction, which translates in NS2 dot formation. Removing the charges would shift the interaction equilibrium and would affect the virus production kinetics in different extents depending on the number of charges and virus strain. Hence, if serine 457 is replaced, the virus titers are moderately reduced at 72h. In contrast, removing serine residues 452, 454, 457 determines a profound defect in infectious virus production. However, there is no NS2 accumulation for either of the two mutants. Thus, the NS2 dots may represent transition states in the assembly process. Indeed, some mutations may prevent the arrival of dots components to NS5A structures (JFH-ΔTM12-HA, JFH-A41-HA, JFH-Δp7-HA and JFH-ΔTME2-HA). Alternatively, other mutations may block the assembly and stabilize them (JFH-HA-PP, JFH-ΔE1E2-HA). If we combine a mutation from the former category (JFH-ΔTME2-HA) with one from the latter (JFH-ΔE1E2-HA) in the mutant JFH-ΔE1E2TME2-HA, we prevent the NS2 dots formation. This suggests that the formation of NS2 complexes and their arrival to the NS5A structures precede the accumulation of NS2 around the LDs during the assembly process. Furthermore, changes in the phosphorylation state of NS5A could regulate the stability of NS2 dots and virion production efficacy.

**Figure 10:**
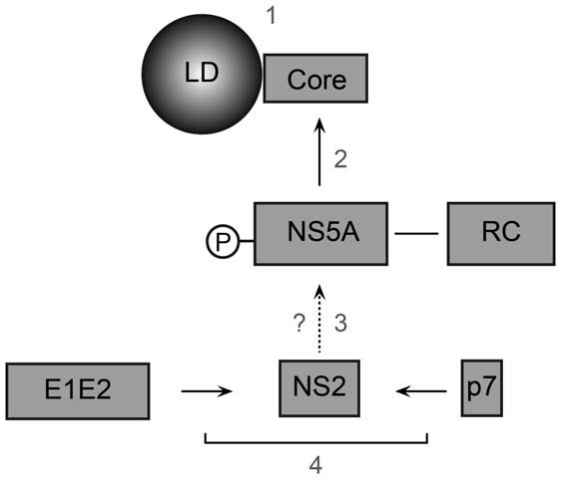
Model of NS2 role in the assembly process. Upon viral polyprotein translation and processing, three viral modules are formed: the core protein (C), the replication complex (RC) and the E1E2p7NS2 complex. E1E2p7NS2 complex assembles through the interaction of E1E2 heterodimer and p7NS2 unit (4) and migrates close to the RC independently of core protein due to signals present in p7NS2 and E2 (3). The core protein localizes around the LDs (1) where it recruits the RC by core-NS5A interaction (2).

In our current view, the assembly process would involve several steps. Upon viral genome translation and polyprotein processing, formation of different complexes occurs: the E1E2 native heterodimer, the p7NS2 unit and the RC. The core protein and other viral proteins (e.g. NS4B) create the LD-ER microenvironment by redistribution of the LDs and intracellular membranes. The LDs surrounded by core protein are recruited to the RC. E1E2 complex interacts with p7NS2 unit and E1E2p7NS2 arrives to NS5A positive membranes in the proximity of LDs due to a combination of signals in p7, NS2 and E2 proteins. NS5A switches from the replication to assembly mode by phosphorylation, which stabilizes the presence of NS2 in dotted structures favoring the assembly process (Figure 10). Finally, our data indicate a crucial role played by NS2 in the assembly process and highlight the complexity of the mechanism of its action. In conclusion, NS2 emerges as an essential mediator between the structural and non-structural proteins in HCV assembly process.

## Materials and Methods

### Cell culture

293T human embryo kidney cells (HEK293T), U2OS human osteosarcoma cells (American Type Culture Collection) and Huh-7 human hepatoma cells [55] were grown in Dulbecco's modified essential medium (Invitrogen) supplemented with 10% fetal bovine serum.

### Antibodies

Anti-HCV Mabs A4 (anti-E1) [56] and 3/11 (anti-E2; kindly provided by J.A. McKeating, University of Birmingham, UK) [57] were produced *in vitro* by using a MiniPerm apparatus (Heraeus) as recommended by the manufacturer. Anti E2 Mab AP33 was kindly provided by A. H. Patel, University of Glasgow, UK. Anti-capsid ACAP27 [58] and anti-NS3 (486D39) Mabs were kindly provided by JF Delagneau (Bio-Rad, France). The anti-NS5A Mab 9E10 [8] and polyclonal antibody were kindly provided by CM Rice (Rockefeller University, NY, USA) and M Harris (University of Leeds, UK), respectively. The anti-NS2 Mab 6H6 [18] and polyclonal antibody were kindly provided by CM Rice (Rockefeller University, NY, USA) and R Bartenschlager (University of Heidelberg, Germany), respectively. The anti-Sec31 antibody [38] was kindly provided by F Gorelick (Yale University School of Medicine, CT, USA). The anti-p125 Mab [37] was kindly provided by K Tani (University of Tokyo, Japan). The following antibodies were purchased: the anti-ERGIC-53 Mab (Alexis), the anti-actin (Santa Cruz Biotechnology), anti-calnexin polyclonal (Stressgen), anti-calreticulin polyclonal (Stressgen), anti-PDI (Stressgen), anti-GFP (Roche) and the anti-hemagglutinin (HA) Mab 3F10 (Roche) and Mab HA11 (Covance). Alexa 488, Alexa 555 and Alexa 633 conjugated goat anti–rabbit, anti-rat and anti-mouse immunoglobulin G (IgG) were purchased from Invitrogen.

### Plasmids

All plasmids were constructed by standard molecular biology methods and the constructs were confirmed by sequencing. The following plasmids were assembled for FRET-FLIM analyses in the background of pCMV plasmid (Addgene): pCMV/YFP-E1TM, pCMV/CFP-E2TM, pCMV/CFP-p7, pCMV/CFP-EYF, pCMV/YFP-NS2. For these constructs, the plasmids encoding YFP and CFP were obtained from DW Piston (Vanderbilt University, USA) and A Miyawaki (Riken Institute, Japan), respectively. YFP-E1TM and CFP-E2TM are YFP and CFP in fusion with the transmembrane domain of E1 and E2 (H strain), respectively. In these two constructs, a two amino acid linker (serine and glycine) was inserted between the fluorescent protein and the transmembrane domains. CFP-p7 and CFP-EYF are CFP proteins fused to the N-terminus of p7 and the transmembrane domain of yellow fever virus envelope protein E (EYF), respectively. YFP-NS2 is a YFP protein fused to the N-terminus of NS2. In all the constructs, the calreticulin signal sequence was fused to the N-terminus of CFP and YFP for translocation in the ER lumen, allowing the study of the recombinant proteins in their native topology.

For p7-NS2 co-immunoprecipitation experiments, the following plasmids were constructed in the background of pTriex (Novagen) or pCI plasmids (Promega): pTriex/p7-Flag, pTriex/EYF-Flag, pCI/HA-NS2, pCI/HA-NS2-GFP and pCI/HA-NS2-GTM. p7-Flag and EYF-Flag are HCV p7 of genotype 1a (H strain) and YFE in fusion with the Flag epitope (DYKDDDDK). HA-NS2 has a HA epitope fused to the N-terminus of NS2. In HA-NS2-GFP, the cytosolic domain of NS2 was replaced by GFP protein, whereas for HA-NS2-GTM, we replaced the transmembrane domain of NS2 by the transmembrane domain of VSV-G protein.

In this work, we used a modified version of the plasmid encoding JFH1 genome (genotype 2a; GenBank access number AB237837), kindly provided by T. Wakita (National Institute of Infectious Diseases, Tokyo, Japan) [7]. Mutations were introduced in a JFH1 plasmid containing a *Renilla* Luciferase reporter gene [59] and mutations leading to amino acids changes F172C and P173S which have been shown to increase the viral titers [30]. Furthermore, the E1 sequence encoding residues ^196^TSSSYMVTNDC has been modified to reconstitute the A4 epitope (SSGLYHVTNDC) [56] as described [31]. Overlapping PCR was used to construct all the JFH1 mutants. The JFH_GND_-Luc construct was obtained by inserting the previously described GND mutation [7] in JFH-Luc plasmid. The JFH-HA construct was obtained by inserting the sequence of the HA epitope (YPYDVPDYA) followed by a GGG linker at the N-terminus of NS2. The JFH-Δp7-HA keeps the first 2 amino acids of p7 followed by the HA tag sequence and the SGG linker at the N-terminus of NS2. The JFH-ΔE1E2-Luc plasmid has been described previously [31]. It contains an in-frame deletion of amino acids 217–567. JFH-ΔTM12-HA has the first two transmembrane segments of NS2 deleted as described [19], in the context of our JFH-HA virus. JFH-HA-PP has proline residues 138 and 143 in domain D2 of the core protein replaced by alanine residues as described [13], [41]. JFH-S/A-HA has serine 457 of NS5A replaced by an alanine as described [17]. JFH-3BS/A-HA has serine residues at positions 452, 454 and 457 of NS5A replaced by alanine residues as described [16]. The JFH-S/D-HA has serine 457 of NS5A replaced by an aspartate residue as described [17]. JFH-ΔTME2-HA and JFH-ΔE1E2TME2-HA have the transmembrane region of E2 glycoprotein replaced by FMDV 2A autoprotease. JFH-ΔTME2-HA contains an in-frame deletion of amino acids 720–750, corresponding to the transmembrane domain of E2 of JFH1, whereas JFH-ΔE1E2TME2-HA contains in-frame deletions of amino acids 217–567 and 720–750. To construct the JFH-ΔTME2-HA, we replaced the C-terminal region of E2 (aa 720–750 of JFH1) by QLLNFDLLKLAGDVESNPGP FMDV 2A autoprotease peptide preceded by a GGG linker sequence. A similar strategy was used for the construction of JFH-ΔE1E2TME2-HA using as a backbone plasmid the JFH-ΔE1E2-HA. JFH-RR/QQ-HA has the arginines 33 and 35 of p7 replaced by glutamine residues. The primers and enzymes used for the constructs are presented in Table S1. Schematic representation of the constructs used in this study is presented in Figure S1.

### DNA transfections

Twenty-four hours before transfection, 293T cells were seeded in 100 mm tissue culture plates to reach a 70–80% confluency the next day. Cells were tranfected with 6 µg of DNA/plate at a ratio of 1∶4 with PEI transfection reagent (Eurogentec). In cotransfection experiments, equal quantities of each plasmid were used. At 24h post-transfection, cells were processed for co-immunoprecipitation analyses.

Twenty-four hours before transfection, U2OS cells were seeded in 6 well plates on 32mm slides to reach a confluency of 70–80% the next day. Cells were transfected with 1µg of CFP-expressing plasmid (donor) and 125 ng of YFP-expressing plasmid (acceptor) mixed with Fugene reagent (Roche) following the instructions of the manufacturer. For CFP-E1 and YFP-E2 co-transfection experiments, we used 300 ng of donor and 600 ng of acceptor plasmids, respectively.

### Two-photon fluorescence lifetime microscopy and data analysis (FRET-FLIM)

Twenty-four hours after transfection, U2OS cells were selected for FRET-FLIM acquisition. We analyzed cells with similar expression levels of donor and acceptor fusion proteins. We also chose cells with normal reticulate ER morphology avoiding those where the overexpression of recombinant proteins was present.

In order to detect the FRET events, the Time Correlated Single Photon Counting FLIM system (TCSPC) was used [60], [61], [62]. The analyses were performed with a Leica SP5.X confocal Microscope (Leica Microsystem) with an internal FLIM detector. A dedicated photo-counting and timing electronic card (SPC 830 TCSPC card, Becker and Hickl) was coupled to the Leica internal detector and used to classify the photon emission in time to determine the lifetime of the donor protein. To excite the samples, Chameleon Ultra2 (Chorent Inc) biphoton was used at 830 nm at an average power of 0.13 mW/µm2 [60], [61], [62].

The fluorescence events of the donor protein result in a photon decay curve generated by the FLIM method. The decay curve was directly used to determine the donor's lifetime. The least square fitting method was used to describe the non linear responses commonly observed in FLIM analysis [60], [61], [62]. We used TITAN (“in the house” designed) and SPCImage (Becker and Hickl) software for advanced FLIM data analysis and curve fittings. In order to reduce the impact of background and improve the Signal to Noise Ratio (SNR), we excluded from our analysis the pixels located in the nuclear region or from ER-like regions where the donor protein was overexpressed. After setting these thresholds, we made a summation of all the pixels of interest to achieve a fitting statistically significant for the TITAN software. To compensate the possible large scattering of points in the curve, we used a Newton trust region algorithm [63] and an extraction of mean lifetimes was performed in order to determine the FRET events from the multi-exponential model [64].

### Immunoprecipitation

Cell pellets were lysed in phosphate-buffered saline (PBS) lysis buffer (1% Triton 100-X, 20mM NEM, 2mM EDTA, protease inhibitors cocktail Roche) and they were precleared with 20 µl Prot G for 2h at 4C. The precleared lysates were incubated with anti-HA antibodies (HA11) or Sepharose beads covalently bound to HA11 antibody (Covance) overnight at 4h. The immunocomplexes were pulled down with 50 µl of Protein G and washed three times with lysis buffer.

For p7-NS2 interaction, cell lysates were incubated with 20 µl of agarose-anti-Flag beads over night at 4°C. The immunocomplexes were treated the same as above and the Western blots were revealed by an anti-HA antibody.

### PNGase and EndoH digestion

The endoglycosidase digestions were performed following the manufacture's instructions (NEB). Briefly, cell lysates containing 20 µg of protein were denatured in EndoH (PNGase) denaturing buffer (0.5% SDS, 1% 2-mercaptoethanol) for 10 min at 100°C. Then, the lysates were incubated or not with 1 µl of EndoH (PNgase) for 20h at 37°C.

### Western blotting

After separation by SDS-PAGE, proteins were transferred to nitrocellulose membranes (Hybond-ECL, Amersham) by using a Trans-Blot apparatus (Biorad) and revealed with specific antibodies followed by secondary immunoglobulin conjugated to peroxidase. The proteins of interest were revealed by enhanced chemiluminescence detection (ECL, Amersham) as recommended by the manufacturer.

### Replication and infectivity assays

Plasmids encoding wild-type (WT) and mutated genomes were linearized at the 3′ end of the HCV cDNA with the restriction enzyme XbaI and treated with the Mung Bean Nuclease (New England Biolabs). *In vitro* transcripts were generated using the Megascript kit according to the manufacturer's protocol (Ambion). The *in vitro* reaction was set up and incubated at 37°C for 4 h and transcripts were precipitated by the addition of LiCl. Ten micrograms of RNA were delivered into Huh-7 cells by electroporation as described previously [30]. Replication was assessed at 72 h by measuring *Renilla* Luciferase activities in electroporated cells as indicated by the manufacturer (Promega). Supernatants containing HCVcc were harvested 72 h after electroporation and filtered through 0.45 µm pore-sized membrane for infectivity measurements. HCVcc were incubated for 3 h with Huh-7 cells seeded the day before in 24-well plates. At 72 h post-infection, Luciferase assays were performed on infected cells as indicated by the manufacturer (Promega). For supernatants titration, Huh7 electroporated cells were seeded in 6-well plates. 72h post-electroporation, naïve Huh-7 cells were inoculated with serial dilutions of the supernatant. 48h post-inoculation, the infected cells were fixed in ice-cold methanol, they were immunostained with anti-E1 antibody and the focus forming units (FFUs) were counted.

### HCV Core quantification

HCV Core was quantified by a fully automated chemiluminescent microparticle immunoassay according to manufacturer's instructions (Architect HCVAg, Abbott, Germany) [65], [66]. For the determination of intracellular core quantity, the electroporated cells were lysed in PBS lysis buffer (1% Triton 100-X, 20mM NEM, 2mM EDTA, protease inhibitors cocktail Roche) and the lysates were cleared for 20 min at 14,000g.

### HCV RNA quantification

Mutated HCV genomes were delivered into Huh-7 cells. At day 3 post-electroporation, cells were trypsinized, washed once with fresh medium and reseeded into cell culture dishes. At day 5 post-electroporation, total RNA in cell lysates and HCV RNA in supernatants were extracted using the Qiagen RNeasy kit and Qiagen QiaAmp viral RNA mini kit, respectively. cDNA was synthesized using High Capacity cDNA Reverse Transcription kit as described by the manufacturer (Applied BioSystems) and titrated by quantitative real-time RT-PCR assay (RT-qPCR) using TaqMan and minor groove binding (MGB) probe detection. The primer pair and the probe were located in the 5′ HCV non-coding region [67].

### Immunofluorescence

Huh-7 cells transfected with HCV RNA were grown on 12-mm glass coverslips. At the indicated time points, cells were fixed with 3% paraformaldehyde and then permeabilized with 0.1% Triton X-100 in PBS. Both primary- and secondary-antibody incubations were carried out for 30 min at room temperature with PBS containing 10% goat serum. LDs were stained for 10 minutes in 300 ng/ml BODIPY 493/503 (Invitrogen). Nuclei were stained with 4,6′-diamidino-2-phenylindole (DAPI). The coverslips were mounted on slides by using Mowiol 4–88 (Calbiochem) containing mounting medium. Confocal microscopy was performed with an LSM710 laser-scanning confocal microscope (Zeiss) using a 63×/1.4 numerical aperture oil immersion objective. Signals were sequentially collected by using single fluorescence excitation and acquisition settings to avoid crossover. Images were processed using Adobe Photoshop software. Cells showing NS2/NS5A-positive dot-like structures were counted on images collected with a 40× oil immersion objective.

### Immunoelectron microscopy

Huh-7 cells transfected with HCV RNA were grown on 75 cm^2^ flasks. At 72h post-electroporation, cells were fixed by incubation in a solution containing 4% paraformaldehyde in 0.1 M phosphate buffer (pH 7.2) for 20 h. The cells were collected by centrifugation and the cell pellet was then dehydrated in a graded series of ethanol solutions at −20°C, using an automatic freezing substitution system (AFS, Leica), and embedded in London Resin Gold (LR Gold, Electron Microscopy Science). The resin was allowed to polymerize at −25°C, under UV light, for 72 h. Ultrathin sections were cut and blocked by incubation with 3% fraction V bovine serum albumin (BSA, Sigma) in PBS. They were then incubated with anti-HA Mab (Covance) diluted 1∶50 in PBS supplemented with 1% BSA, washed and incubated with goat anti-mouse antibodies conjugated to 15 nm gold particles (British Biocell International, Cardiff, UK) diluted 1∶40 in PBS supplemented with 1% BSA. Ultrathin sections were cut, stained with 5% uranyl acetate, 5% lead citrate, and placed on electron microscopy grids coated with collodion. The sections were then observed with a Jeol 1230 transmission electron microscope (Tokyo, Japan) connected to a Gatan digital camera driven by Digital Micrograph software (Gatan, Pleasanton, CA) for image acquisition.

## Supporting Information

Figure S1 Mutations in NS2 affect viral assembly. (A) Schematic representation of the constructs used in this study. (B) Characterization of the phenotypes of HCV mutants. Huh7 cells were electroporated with the indicated genomes. At 72h post-electroporation supernatants were collected and cells were lysed. Replication was determined as the ratio between Rluc counts at 72h and 4h, respectively (see Replication panel). In parallel, virus-producing cells were washed and lysed by repetitive cycles of freeze and thaw. Extracellular (black bars) and intracellular (white bars) infectivities were determined by inoculating naïve cells and measuring the Rluc activities at 72h post-inoculation (see Infectivity panel). To measure viral secretion, mutated HCV genomes were delivered to Huh-7 cells. At two days post-electroporation, cells were trypsinized, washed once and reseeded into cell culture dishes. HCV RNA levels in cell lysates and in supernatants were extracted 5 days after electroporation, and titrated by quantitative real-time RT-PCR (see Secretion panel). Viral secretion was considered equivalent to the genomic viral RNA released in the media. Error bars indicate SD from at least two independent experiments. (C) NS2 mutants protein stability. Huh-7 cells were electroporated with viral RNA transcribed from different JFH-1 derived mutants. At 72h post-electroporation, cells were lysed, separated by SDS-PAGE and analyzed by Western blotting with an anti-E2, anti-HA or anti-NS2. The actin content was also analyzed to verify that equal amounts of cell lysates have been loaded.
(3.05 MB TIF)

Figure S2 NS2 colocalization with the viral proteins. JFH-HA electroporated cells grown on coverslips were fixed at 72h post-electroporation and processed for double-label immunofluorescence for HA-NS2 (red) and HCV proteins core (C), NS5A or E1 (green). The nuclei were stained with DAPI (blue). Representative confocal images of individual cells are shown in grey and the colored merge images in the right column. Insets display zoomed views of the indicated area. Bar, 10 µm.
(5.85 MB TIF)

Figure S3 Alternative subcellular localization of NS2. JFH-HA electroporated cells grown on coverslips were fixed at 72h post-electroporation and processed for double-label immunofluorescence for HA-NS2 (Red) and HCV core (C), ER-to-Golgi intermediate compartment marker ERGIC-53, ER exit site marker sec31, or Golgi marker GM130 (Green). Nuclei were stained with DAPI (Blue). Representative confocal images of individual cells are shown with the merge images in the right column. Cells showing NS2 alternative subcellular localization are indicated by a star. Note the difference of ERGIC-53 and GM130 patterns in cells showing NS2 alternative subcellular localization. Bar, 10 µm.
(8.33 MB TIF)

Figure S4 NS2 subcellular localization for JFH-HA-PP and ΔE1E2 mutants. (A) Huh-7 cells electroporated with JFH-HA RNA (WT) or JFH-HA-PP (PP) or JFH-ΔE1E2-HA (ΔE1E2) genomes were grown on coverslips, fixed at 72h post-electroporation and processed for triple-label immunofluorescence for HA-NS2 (red), NS5A (blue) and LD (green). The nuclei were stained with DAPI (grey). (B) E1-immunoreactive material (green) was analyzed with the A4 Mab, together with HA-NS2 (red) for the ΔE1E2 mutant and JFH-HA (WT). The nuclei were stained with DAPI (blue). Representative confocal images of individual cells are shown in grey with the colored merge images in the right column. Bar, 10 µm.
(9.03 MB TIF)

Figure S5 The biphoton pictures for the positive and negative control of FRET-FLIM analysis. U2OS cells were co-transfected with plasmids expressing CFP-EYF and YFP-NS2 (negative control, panel B) or CFP-E2TM and YFP-E1TM (positive control, panel A). At 24h post-transfection, samples were subjected to FLIM and color coded maps were obtained. The color bar represent the progression from minimum (yellow) to maximum (blue) fluorescence lifetime.
(2.02 MB TIF)

Table S1 Primers used for constructs.
(0.04 MB DOC)
