# Supplementary figures and images for: NS2 Protein of Hepatitis C Virus Interacts with Structural and Non-Structural Proteins towards Virus Assembly
Source: PLoS Pathog. 2011 Feb 10;7(2):e1001278. doi: 10.1371/journal.ppat.1001278 (PMC3037360; doi:10.1371/journal.ppat.1001278)

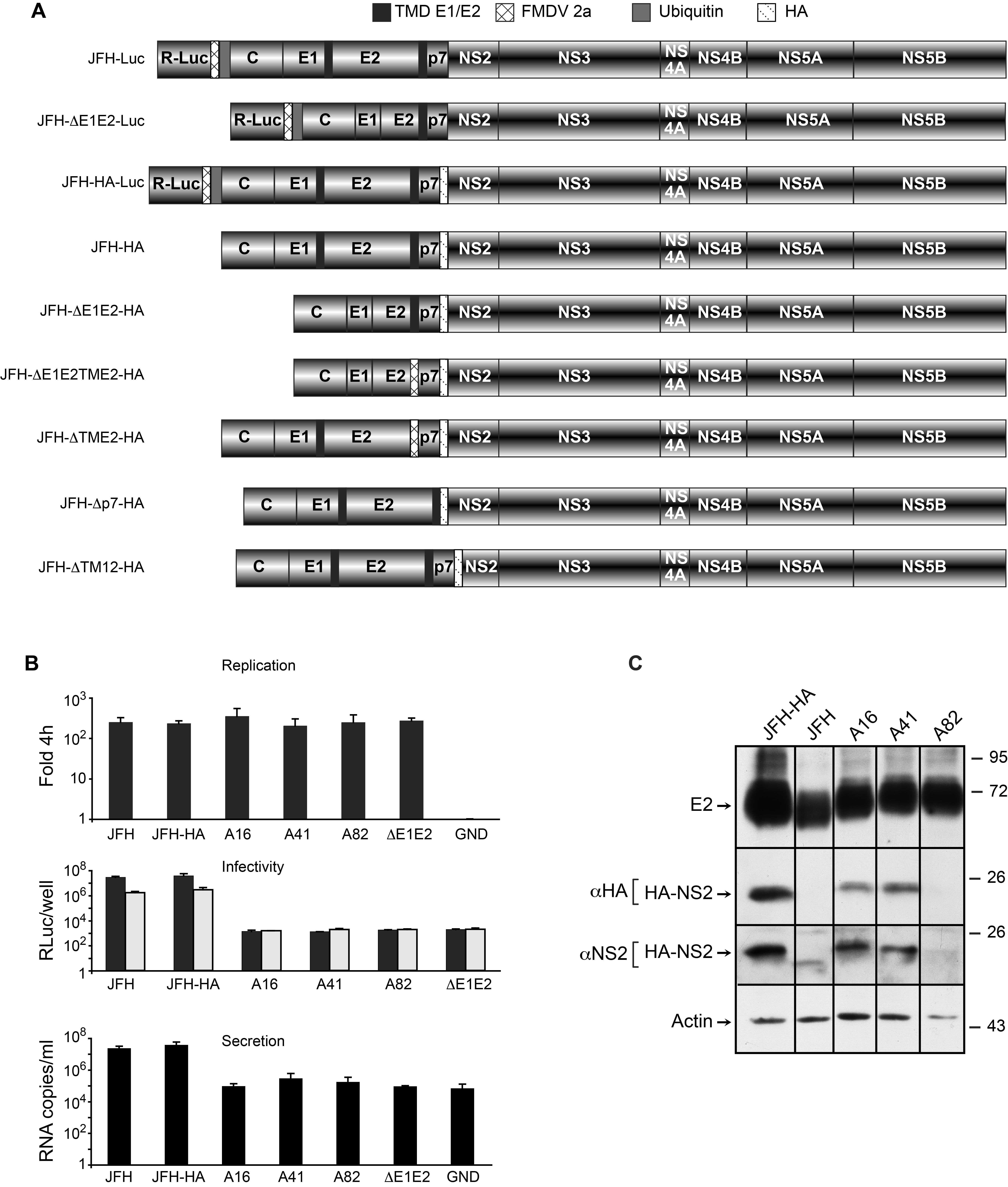

Supplement: Figure S1 — Mutations in NS2 affect viral assembly. (A) Schematic representation of the constructs used in this study. (B) Characterization of the phenotypes of HCV mutants. Huh7 cells were electroporated with the indicated genomes. At 72h post-electroporation supernatants were collected and cells were lysed. Replication was determined as the ratio between Rluc counts at 72h and 4h, respectively (see Replication panel). In parallel, virus-producing cells were washed and lysed by repetitive cycles of freeze and thaw. Extracellular (black bars) and intracellular (white bars) infectivities were determined by inoculating naïve cells and measuring the Rluc activities at 72h post-inoculation (see Infectivity panel). To measure viral secretion, mutated HCV genomes were delivered to Huh-7 cells. At two days post-electroporation, cells were trypsinized, washed once and reseeded into cell culture dishes. HCV RNA levels in cell lysates and in supernatants were extracted 5 days after electroporation, and titrated by quantitative real-time RT-PCR (see Secretion panel). Viral secretion was considered equivalent to the genomic viral RNA released in the media. Error bars indicate SD from at least two independent experiments. (C) NS2 mutants protein stability. Huh-7 cells were electroporated with viral RNA transcribed from different JFH-1 derived mutants. At 72h post-electroporation, cells were lysed, separated by SDS-PAGE and analyzed by Western blotting with an anti-E2, anti-HA or anti-NS2. The actin content was also analyzed to verify that equal amounts of cell lysates have been loaded. (3.05 MB TIF) [file ppat.1001278.s001.tif]

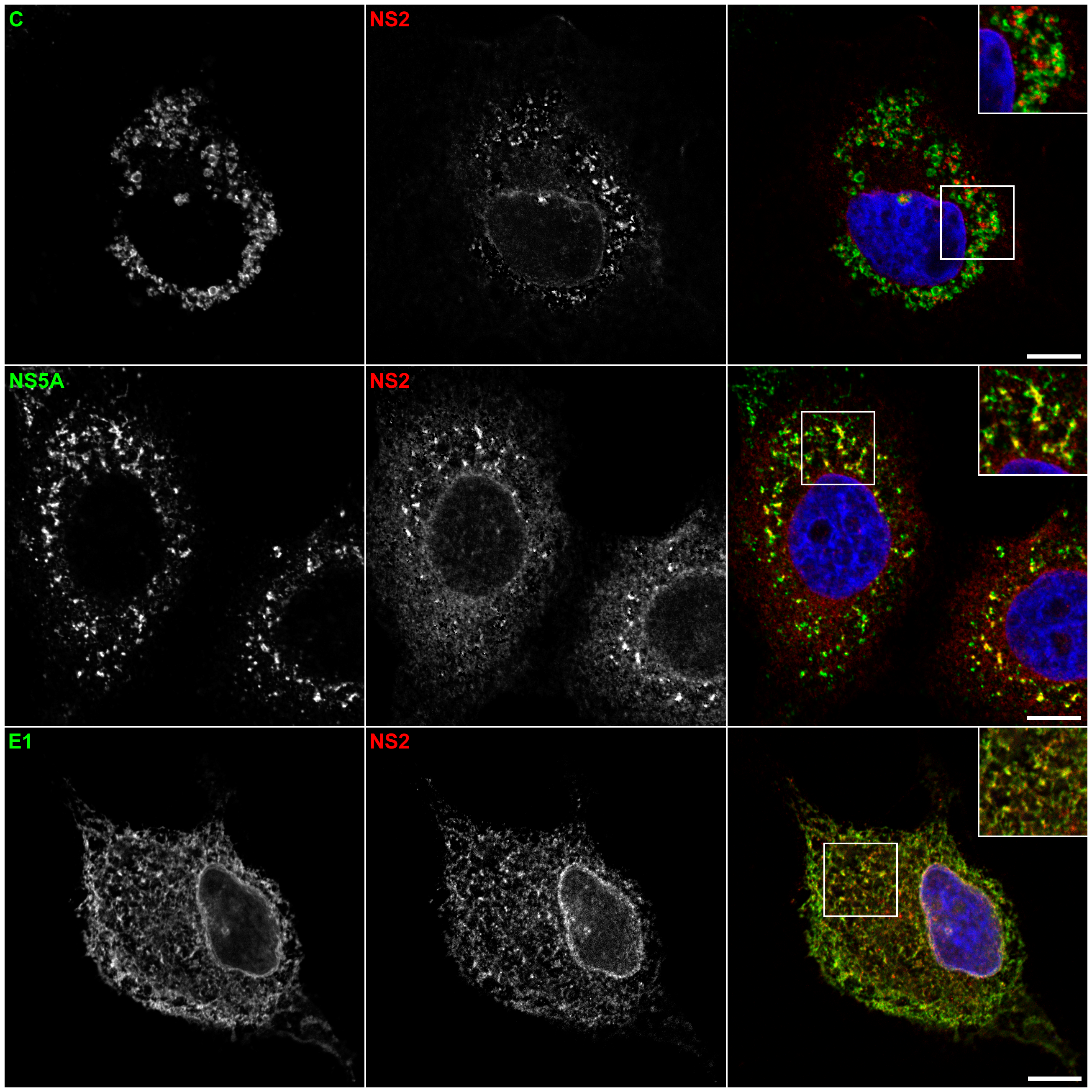

Supplement: Figure S2 — NS2 colocalization with the viral proteins. JFH-HA electroporated cells grown on coverslips were fixed at 72h post-electroporation and processed for double-label immunofluorescence for HA-NS2 (red) and HCV proteins core (C), NS5A or E1 (green). The nuclei were stained with DAPI (blue). Representative confocal images of individual cells are shown in grey and the colored merge images in the right column. Insets display zoomed views of the indicated area. Bar, 10 µm. (5.85 MB TIF) [file ppat.1001278.s002.tif]

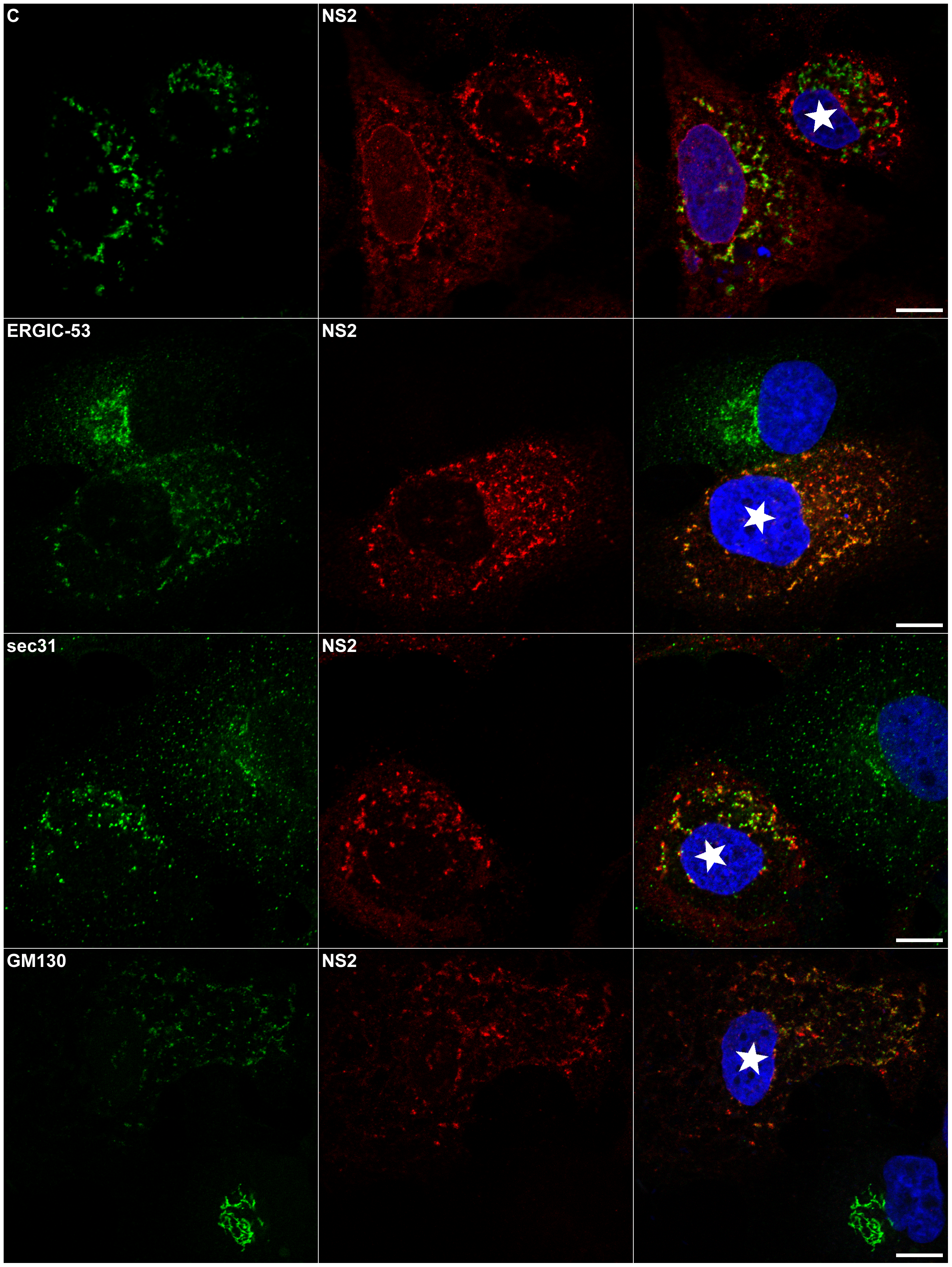

Supplement: Figure S3 — Alternative subcellular localization of NS2. JFH-HA electroporated cells grown on coverslips were fixed at 72h post-electroporation and processed for double-label immunofluorescence for HA-NS2 (Red) and HCV core (C), ER-to-Golgi intermediate compartment marker ERGIC-53, ER exit site marker sec31, or Golgi marker GM130 (Green). Nuclei were stained with DAPI (Blue). Representative confocal images of individual cells are shown with the merge images in the right column. Cells showing NS2 alternative subcellular localization are indicated by a star. Note the difference of ERGIC-53 and GM130 patterns in cells showing NS2 alternative subcellular localization. Bar, 10 µm. (8.33 MB TIF) [file ppat.1001278.s003.tif]

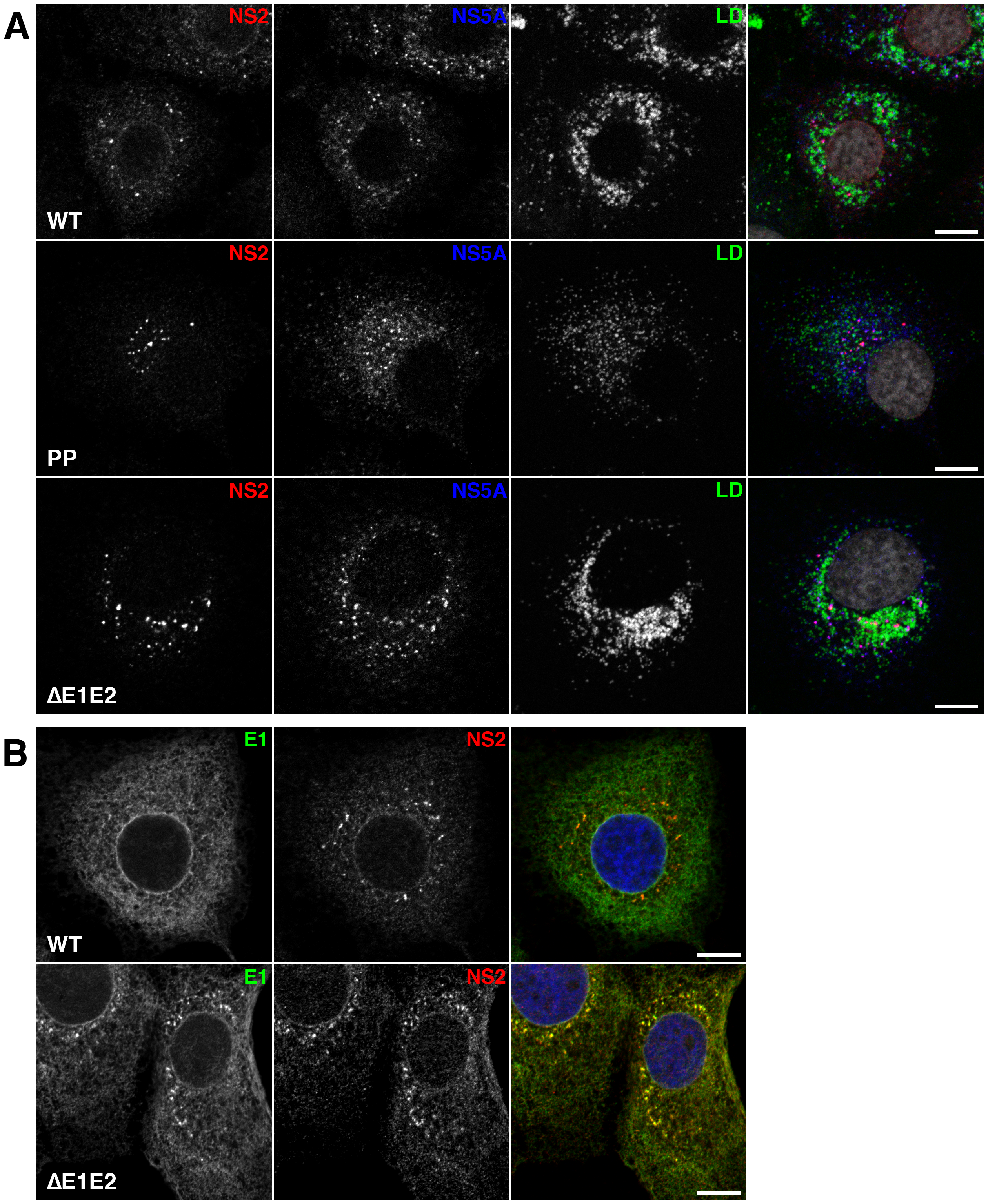

Supplement: Figure S4 — NS2 subcellular localization for JFH-HA-PP and ΔE1E2 mutants. (A) Huh-7 cells electroporated with JFH-HA RNA (WT) or JFH-HA-PP (PP) or JFH-ΔE1E2-HA (ΔE1E2) genomes were grown on coverslips, fixed at 72h post-electroporation and processed for triple-label immunofluorescence for HA-NS2 (red), NS5A (blue) and LD (green). The nuclei were stained with DAPI (grey). (B) E1-immunoreactive material (green) was analyzed with the A4 Mab, together with HA-NS2 (red) for the ΔE1E2 mutant and JFH-HA (WT). The nuclei were stained with DAPI (blue). Representative confocal images of individual cells are shown in grey with the colored merge images in the right column. Bar, 10 µm. (9.03 MB TIF) [file ppat.1001278.s004.tif]

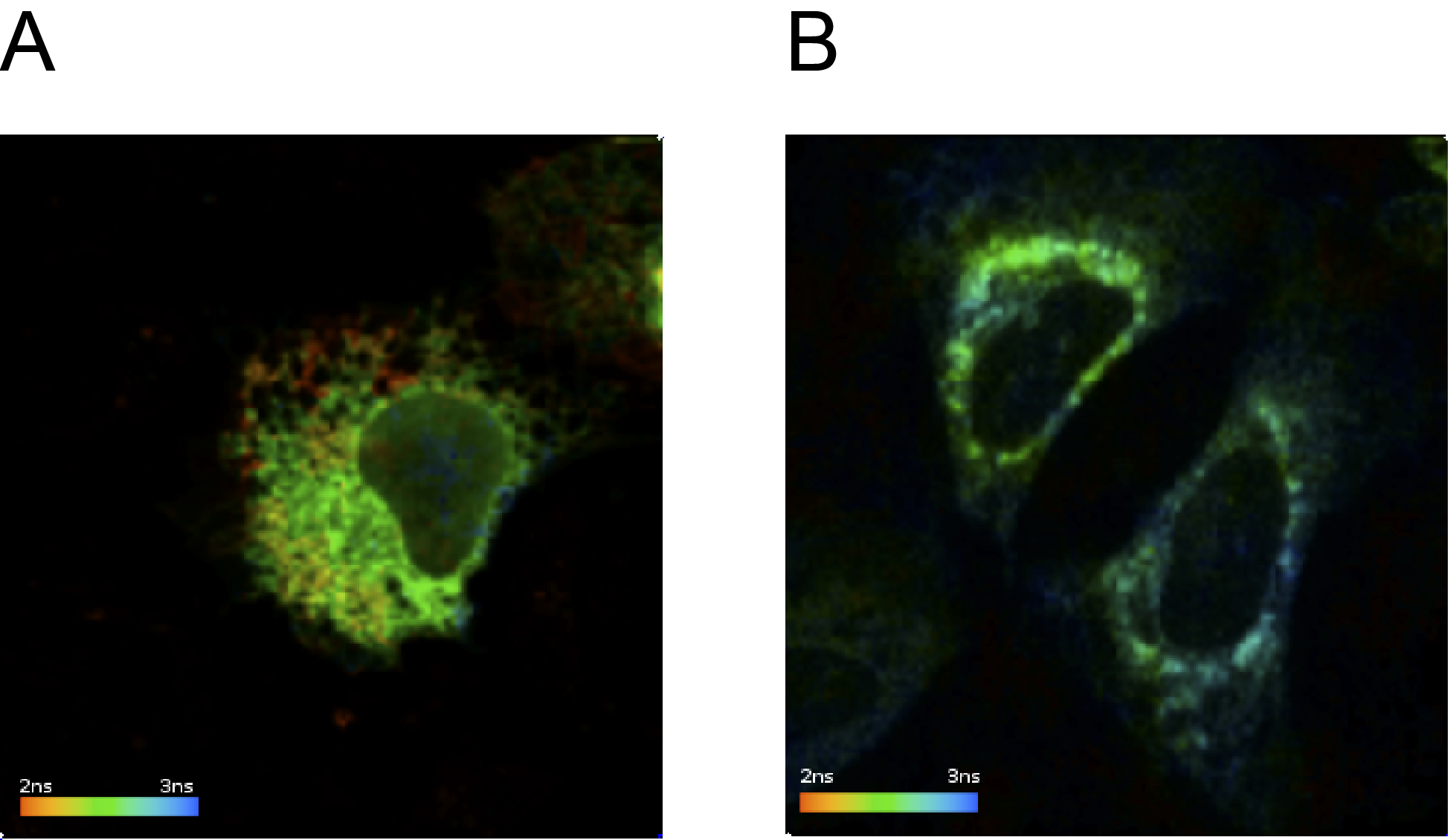

Supplement: Figure S5 — The biphoton pictures for the positive and negative control of FRET-FLIM analysis. U2OS cells were co-transfected with plasmids expressing CFP-EYF and YFP-NS2 (negative control, panel B) or CFP-E2TM and YFP-E1TM (positive control, panel A). At 24h post-transfection, samples were subjected to FLIM and color coded maps were obtained. The color bar represent the progression from minimum (yellow) to maximum (blue) fluorescence lifetime. (2.02 MB TIF) [file ppat.1001278.s005.tif]
