# Supplementary material for: NS2 Protein of Hepatitis C Virus Interacts with Structural and Non-Structural Proteins towards Virus Assembly
Source: PLoS Pathog. 2011 Feb 10;7(2):e1001278. doi: 10.1371/journal.ppat.1001278 (PMC3037360; doi:10.1371/journal.ppat.1001278)
Supplement: Table S1 — Primers used for constructs. (0.04 MB DOC) [file ppat.1001278.s006.doc]

Table S1: primers used for constructs

| Construct name | Primer name | Primer sequence | Enzymes in the flanking primers |
| --- | --- | --- | --- |
| JFH-HA | P1-F  P1-R | GCCCCGGCAGGCTTATGCCTACCCATACGACGTCCCAAGACTACGCTGGCGGAGGATATGACGCACCTGTGCACG  CGTGCACAGGTGCGTCATATCCTCCGCCAGCGTAGTCTGGGACGTCGTATGGGTAGGCATAAGCCTGCCGGGGC | BsiWI/KpnI |
| JFH-∆p7-HA | P2-F  P2-R | CAGGCCGAAGCAGCATTGTACCCATACGACGTCCCAGACTACGCGGGCGGAGGATATGACGCACCTGTG  tcctccggacgcgtagtctgggacgtcgtatgggtaCAATGCTGCTTCGGCCTGGCCCAACAAGATGAG | BsiWI/KpnI |
| JFH-ΔTM12-HA | P3-F  P3-R | CCTGTGCACGGACAGATAGGCATCGCGTGGGCCGTC  GACGGCCCACGCGATGCCTATCTGTCCGTGCACAGG | BsiWI/SpeI |
| JFH−ΔTME2-HA | P4-F  P4-R | ctcaagttggccggcgacgtcgagtccaacccagggcccGCATTGGAGAAGTTGGTCGTC  gtcgccggccaacttgagaaggtcaaagttgccaccgccTCGAACGACGTATTTTGTGATAG | BsiWI/KpnI |
| JFH−ΔE1E2TM-HA | P4-F  P4-R | ctcaagttggccggcgacgtcgagtccaacccagggcccGCATTGGAGAAGTTGGTCGTC | AleI/KpnI |
